# Sex differences in modifiable risk factors of dementia and their associations with cognition

**DOI:** 10.1186/s13293-026-00908-7

**Published:** 2026-05-20

**Authors:** Megan C. Fitzhugh, Judy Pa

**Affiliations:** 1 Department of Neurosciences, University of California, 9500 Gilman Drive, MC 0949, La Jolla, San Diego, CA 92093-0949 USA; 2 Alzheimer’s Disease Cooperative Study (ADCS), University of California, La Jolla, San Diego, CA 92093 USA

**Keywords:** Sex differences, Modifiable risk factors, Cognition, Prevalence, Dementia

## Abstract

**Background:**

Women have a greater lifetime risk of developing dementia. Despite clear sex differences, studies investigating modifiable dementia risk factors often overlook differences between sexes and age. This study examined sex and age differences in the prevalence of modifiable dementia risk factors and their associations with cognition.

**Methods:**

Participants were from the Health and Retirement Study, a nationally representative cohort study. Thirteen risk factors were examined, including education, hearing loss, cholesterol, depression, physical inactivity, diabetes, smoking, hypertension, obesity, excessive alcohol use, social isolation, poor vision, and poor sleep. A global cognitive summary score was also examined. Chi-square and t-tests examined sex and age differences in prevalence; linear regression examined interactions between sex, age, and risk factors on cognition.

**Results:**

This study included 17,182 participants with a mean age of 69.2 ± 10.6 years, 59.2% of which were women. Ten out of 13 risk factors had sex differences in prevalence. Women had higher prevalence of elevated cholesterol, depression, physical inactivity, smoking, poor vision, and poor sleep. Women also had fewer years of education. Men had a higher prevalence of hearing loss, diabetes, and excessive alcohol use. Hearing loss, diabetes, and hypertension were associated with greater effects on cognitive performance in woman than men. BMI was negatively associated with cognitive performance in women compared to men in their 50s and 60s, but not at older ages. Education and cholesterol had stronger, positive associations with cognitive performance in women compared to men.

**Conclusions:**

These data suggest that women’s greater risk of dementia may be due to a higher prevalence of multiple risk factors and stronger cognitive effects of risk factors. Results may inform future personalized prevention strategies for dementia risk reduction, particularly in women.

**Supplementary Information:**

The online version contains supplementary material available at 10.1186/s13293-026-00908-7.

## Abstract (plain language summary)

Women are more likely than men to develop dementia over their lifetime, but many studies do not examine how dementia risk factors differ by sex or age. This study investigated whether modifiable dementia risk factors are more common in women or men and whether these risk factors differentially impact cognition by sex. Thirteen risk factors linked to dementia were examined: education, hearing loss, cholesterol, depression, physical inactivity, diabetes, smoking, high blood pressure, obesity, alcohol use, social isolation, poor vision, and poor sleep. Women had a higher prevalence of more risk factors than men, including poor sleep, poor vision, physical inactivity, cholesterol, depression, smoking, and fewer years of education. Men were more likely to have hearing loss, diabetes, and high alcohol use. Several risk factors were linked to worse cognitive performance in women than men, including hearing loss, diabetes, hypertension, and obesity. However, more years of education was associated with better cognition in women compared to men. These findings suggest that women’s higher dementia risk may reflect both greater exposure to risk factors and stronger negative effects of these risk factors on cognition.

**Supplementary Information:**

The online version contains supplementary material available at 10.1186/s13293-026-00908-7.

## Highlights

- Women had higher prevalence in 7 of 13 examined modifiable risk factors.
- Men had higher prevalence in only 3 of the 13 risk factors.
- 3 risk factors (hearing loss, diabetes, and hypertension) had larger, negative effects on cognition in women compared to men.
- 2 risk factors (cholesterol and education) had larger, positive effects on cognition in women compared to men.

**Supplementary Information:**

The online version contains supplementary material available at 10.1186/s13293-026-00908-7.

## Background

Alzheimer’s disease is a major public health priority, affecting one in nine adults aged 65 and older in the United States [1]. Of the seven million adults living with Alzheimer’s disease, nearly two-thirds are women [2]. Not only is Alzheimer’s disease more prevalent in women, the lifetime risk of developing dementia due to Alzheimer’s disease is 20% for women and only 10% for men in the US [3], with similar findings across Europe [4]. However, other studies report no sex differences in the incidence of Alzheimer’s disease [5, 6], suggesting that sex differences may depend on age or the cohort examined. While on average, women live longer than men, differences in longevity do not fully explain the higher prevalence and increased risk of Alzheimer’s disease experienced by women [7].

It has been suggested that sex differences in Alzheimer’s disease and related dementias may be explained, in part, by differences in the influence of modifiable risk factors. The Lancet Commission on Dementia Prevention, Intervention, and Care and others have identified 14 risk factors that over the life course accumulate and contribute to dementia risk [8, 9]. If these risk factors are mitigated or eliminated, it could reduce an estimated 45% of dementia cases. The Lancet Commission further identifies the period in the lifespan at which each risk factor has its greatest effect on dementia risk, including early life (education), midlife, between the ages of 45 and 65 (hearing loss, LDL cholesterol, depression, traumatic brain injury, physical inactivity, smoking, diabetes, hypertension, obesity, and excessive alcohol use), or late life, after age 65 (social isolation, air pollution, and vision loss).

There is a growing recognition for the need to develop risk reduction approaches that target one or more modifiable risk factors of dementia, including interventions like the US POINTER [10], World-Wide FINGERS [11] and SMARRT [12, 13] trials, and educational platforms like HALT-AD [14] and Brain Health PRO [15, 16]. However, these efforts often do not consider sex as part of the individualized intervention. Evidence of sex differences across numerous dementia risk factors and their impact on dementia risk is a small but growing body of research. Findings from observational studies show that women and men express modifiable risk factors of dementia at different rates. This work suggests that women experience high cholesterol, physical inactivity, and depression in greater numbers than men, more men experience diabetes, hearing loss, and substance abuse (including smoking and alcohol), and sex differences in the prevalence of hypertension varies by study [17–22]. It is a major challenge to change behavior, particularly when asked to change multiple behaviors. Therefore, it is critical that we identify which risk factors to target considering both the individual’s sex and age in order to tailor our interventions to the risk factors that not only have the highest prevalence, but also the greatest potential impact on cognition.

The purpose of this study was to conduct a comprehensive, secondary data analysis to identify sex and age differences in (1) the prevalence of modifiable risk factors of dementia and (2) the association between each risk factor and cognitive performance. This study included over 17,000 participants who were 40 years or older at the 2008 data collection wave of the Health and Retirement Study. Thirteen modifiable risk factors were examined. We hypothesized that men would have a higher prevalence in more risk factors compared to women. However, given women’s greater risk of developing dementia, we predicted that the presence of a given risk factor would have a greater effect on cognitive performance on women compared to men.

## Methods

### Study population

The Health and Retirement Study is a nationally representative, longitudinal study of adults in the United States aged 50 years and older, and their spouses (of any age), at the time of recruitment. The study began in 1992 and collects, in part, comprehensive self-report questionnaires on demographics, health status, functional activities, cognition, occupation and financial status every two years. This study used data from the 2008 collection wave from participants who were 40 years in age and older. It should be noted that beginning in 2006, participants did not complete all questionnaires and assessments at every study wave. Instead, participants were split into two groups and alternated in-depth, in-person data collection by study wave, such that at any study wave only half of the sample completed all assessments [23].

### Consent statement

Participants and their informants provided written informed consent to participate. The study protocols for the Health and Retirement Study and Harmonized Cognitive Assessment Protocol were approved by the University of Michigan Institutional Review Board.

### Risk factors

Information on 12 modifiable risk factors from the 14 identified in the Lancet Commission 2024 report were available within the dataset [8]. These risk factors included years of education, hearing loss, total cholesterol (as a proxy for LDL cholesterol), depression, physical inactivity, diabetes, smoking, hypertension, obesity, excessive alcohol use, social isolation, and poor vision. The self-report questionnaires did not include queries for two of the risk factors identified by the Lancet Commission: traumatic brain injury and air pollution. We included poor sleep as a risk factor because it has been repeatedly recognized as a potential risk factor in the Lancet Commission Reports, but currently has insufficient evidence to include in their models [24, 25]. In Additional File 1, we describe how these 13 total risk factors were operationalized from self-report questionnaires or from health data for our study. Briefly, 10 of the 13 risk factors were dichotomized into “Yes, presence of risk factor” or “No, absence of risk factor” from questionnaires, while years of education, total cholesterol, and obesity (calculated as body mass index (BMI)) were left as continuous variables.

To assess sex differences in the influence of multiple risk factors on cognitive performance, two different composite scores were computed. The first summed the presence (Yes = 1, No = 0) of each risk factor, for a total possible composite score of 13. The three continuous risk factors were binarized such that less than a high school education, total cholesterol levels greater than or equal to 200 mg/dL, and BMI greater than or equal to 30 were coded as 1 (Yes, presence of risk factor), with other values coded 0 (No, absence of risk factor). The second composite adjusted for the proportion of variance shared between risk factors using the adjusted population attributable fraction (PAF) for each risk factor [8]. Adjusted risk was calculated by multiplying the PAF of a given risk factor by the risk factor presence (1 = Yes, 0 = No), dividing by the total PAF across all risk factors, and summing across risk factors for a total possible score of 1. While the Lancet Commission on Dementia Prevention identifies an overall PAF across all risk factors of 45%, since we could not operationalize TBI and air pollution in this study, our total PAF was 39%. Sleep was not included as risk factor in these analyses because it does not yet have a PAF score.

### Cognitive performance

Cognitive performance was assessed using a shortened version of the Telephone Interview for Cognitive Status (TICS [26]). This assessment is a brief test of global mental status and includes a 10-word immediate and delayed recall, serial 7 subtractions, and backwards counting from 20. A summary score reflecting performance on each test was previously created by the Health and Retirement Study team. The score was calculated as follows: 10 total points for immediate word recall, 10 points for delayed word recall, five total points for serial 7 s, and up to two total points for backwards counting (two points for correct counting on the first attempt, one point for correct counting on the second attempt, or no points for incorrectly counting on the first and second attempts), for a total score of 27 points [27]. This summary score is the only cognitive performance score assessed in the entire Health and Retirement Study sample, which allowed a large sample size for our scoping assessment of several risk factors. The TICS has been compared against comprehensive neuropsychological batteries and is effective at categorizing cognitive impairment and dementia from those who are cognitively unimpaired [28–30].

### Statistical analysis

To determine if the prevalence or mean value of the risk factors differed by sex, Chi-square tests (for dichotomous risk factors) and Student’s *t*-tests (for continuous risk factors) were used. Sex differences were examined within the whole sample and within age groups. An age group variable was created to separate participants into a less than 65 years in age group, or a 65 years in age or older group, for comparison to the simple effects performed following significant interactions in linear regression models (see below). The threshold of 65 years was chosen to reflect the inflection point between midlife and late life described in the Lancet Commission on Dementia Prevention. Significance was set at *p* <.05 with Bonferroni corrections for multiple comparisons across all tests.

Parallel linear regression models were used to examine the three-way interaction of risk factor, sex, and age on the TICS cognitive summary score. Significant three-way interactions were followed by simple effects (or simple slopes for continuous variables) between risk factor (yes vs. no, the reference) and sex, at three representative ages, 55, 65, and 75 years. These representative ages were selected to reflect the Lancet Commission on Dementia Prevention’s inflection point between midlife and late life, 65 years, and 10 years before and after this point. For risk factors with no significant three-way interactions, models were reduced to include only two-way interactions between risk factor and sex, and were followed up by simple effects or slopes of the risk factor within men and women separately. Parallel models were also run using the unadjusted and adjusted risk factor composite scores as predictors. Significance for the three- and two-way model interactions was set at *p* <.05. Significance for the simple effects was set at *p* <.05 with Bonferroni corrections for multiple comparisons within each risk factor.

Since many of the predictors in our analyses are commonly used as covariates in investigations of cognition in older adults (e.g., age, sex, education, hypertension, etc.), the primary analyses were performed without covariates. Secondary analyses were then performed that included years of education (except for the model where education is the risk factor of interest, which was excluded from the secondary analyses). Significance for tests in the secondary analyses was set at *p* <.05, uncorrected for multiple comparisons.

Chi-square tests and *t*-tests for prevalence comparisons were performed using SPSS (IBM Corp., release 2020, IBM SPSS Statistics for Macintosh, version 28.0.1). Linear regressions were conducted using R (v4.4.1) and RStudio (v2026.01.0) via the {lm} package. Significant interactions were decomposed using {emmeans} and {emtrends} packages.

## Results

### Cohort demographics

**Fig. 1:**
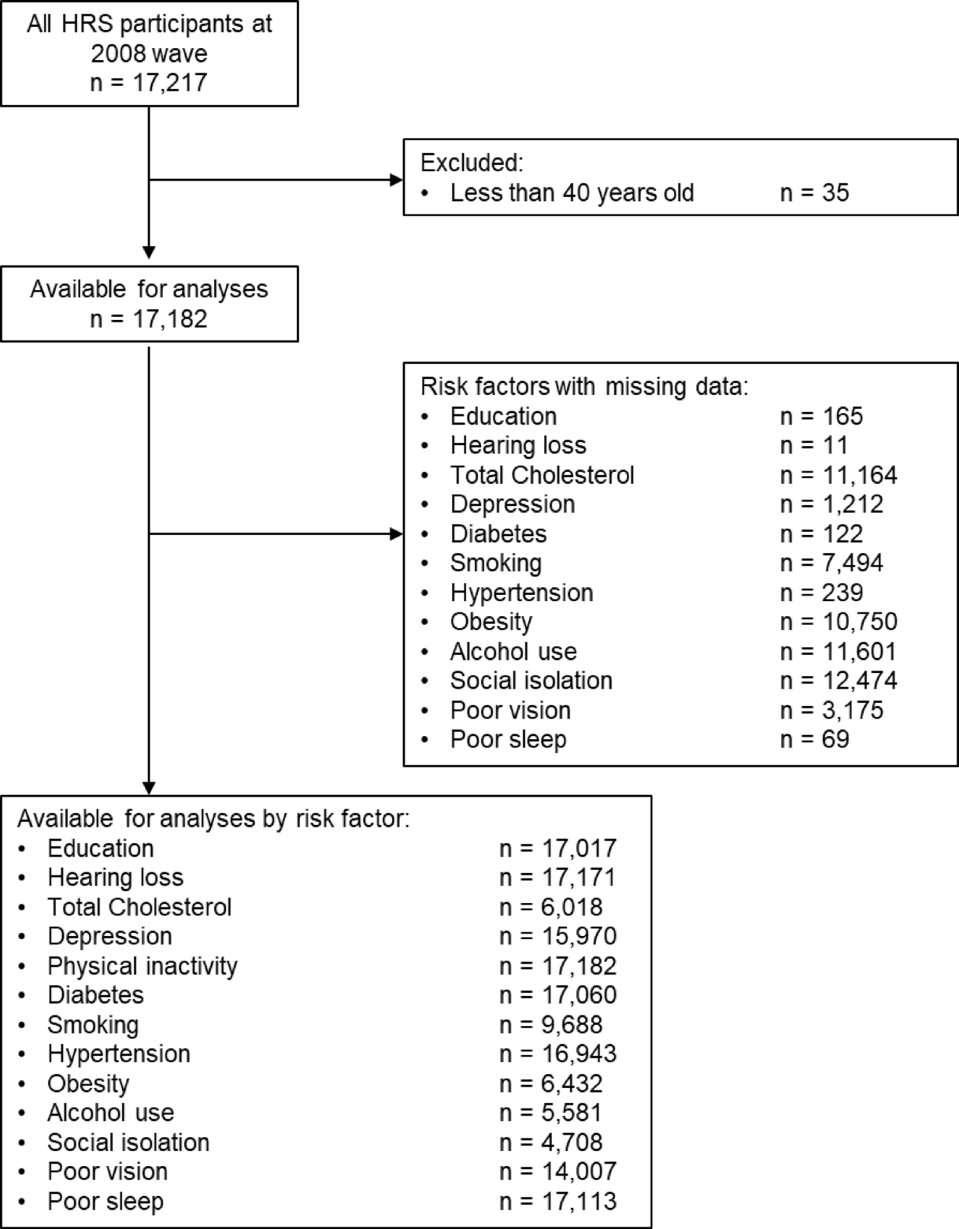
Available participant sample sizes per risk factor. Flowchart of all available participants in the 2008 Health and Retirement Study wave, number of missing data per risk factor, and sample size per risk factor

This study included 17,182 participants who were 40 years of age or older at the 2008 Health and Retirement Study data collection wave (see Fig. 1 for missing data and total sample size for each risk factor). This included 7,009 men (40.8%) and 10,173 women (59.2%). The mean (SD) age was 69.2 (10.7) years, and the mean (SD) total years of education was 12.4 (3.3). Table 1 includes the prevalence (or mean) of each risk factor for the entire study sample and stratified by sex.

**Table 1:** Prevalence (or mean) of 13 modifiable lifestyle risk factors of dementia

	All (*N* = 17,182)	Women (*n* = 10,173)	Men (*n* = 7,009)	Sex Difference
Age (Mean (SD), years) (*n* = 17,182)	69.2 (10.7)	69.1 (11.1)	69.4 (9.9)	*p* <.001*
Education (Mean (SD), years) (*n* = 17,017; w = 10,092; m = 6,925)	12.4 (3.3)	12.3 (3.1)	12.6 (3.5)	*p* <.001*
Poor Hearing No. (% Yes) (*n* = 17,171)	9,605 (56%)	5,105 (50%)	4,500 (64%)	*p* <.001*
Total Cholesterol (Mean (SD)) (*n* = 6,018; w = 3,625; m = 2,393)	201.0 (42.0)	207.1 (42.5)	191.7 (39.3)	*p* <.001*
Depression No. (% Yes) (*n* = 15,970)	2,222 (14)	1,623 (17%)	599 (9%)	*p* <.001*
Physical Inactivity No. (% Yes) (*n* = 17,182)	7,821 (46)	4,908 (48%)	2,913 (42%)	*p* <.001*
Diabetes No. (% Yes) (*n* = 17,060)	3,767 (22)	2,087 (21%)	1,680 (24%)	*p* <.001*
Current Smoker No. (% Yes) (*n* = 9,688)	2,221 (23)	1,278 (26%)	943 (20%)	*p* <.001*
Hypertension No. (% Yes) (*n* = 16,943)	10,383 (61)	6,164 (62%)	4,219 (61%)	*p* =.511
BMI (Mean (SD)) (*n* = 6,432; w = 3,838; m = 2,594)	29.3 (6.1)	29.4 (6.7)	29.2 (5.1)	*p* =.226
Alcohol Use No. (% Yes) (*n* = 5,581)	968 (17)	313 (12%)	655 (22%)	*p* <.001*
Social Isolation No. (% Yes) (*n* = 4,708)	644 (14)	407 (15%)	237 (12%)	*p* =.025
Poor Vision No. (% Yes) (*n* = 14,007)	4,034 (29)	2,495 (30%)	1,539 (28%)	*p* =.009
Poor Sleep No. (% Yes) (*n* = 17,113)	7,379 (43)	4,579 (45%)	2,800 (40%)	*p* <.001*

The prevalence reported as percent of the available sample (or mean) for each risk factor is shown for the entire study sample and within men and women. The sample size available for each risk factor is also provided, as not all participants were asked to partake in each study wave, per the Health and Retirement Study design, and not all participants chose to respond to each question. The p-values are shown unadjusted, but we indicate with a * which results survive correction for multiple comparisons using the Bonferroni method (13 risk factors * 3 tests = 39 separate tests, adjusted alpha 0.05/39=0.001; see Additional File 2 for complete statistics)

### Sex differences in risk factor prevalence

**Fig. 2:**
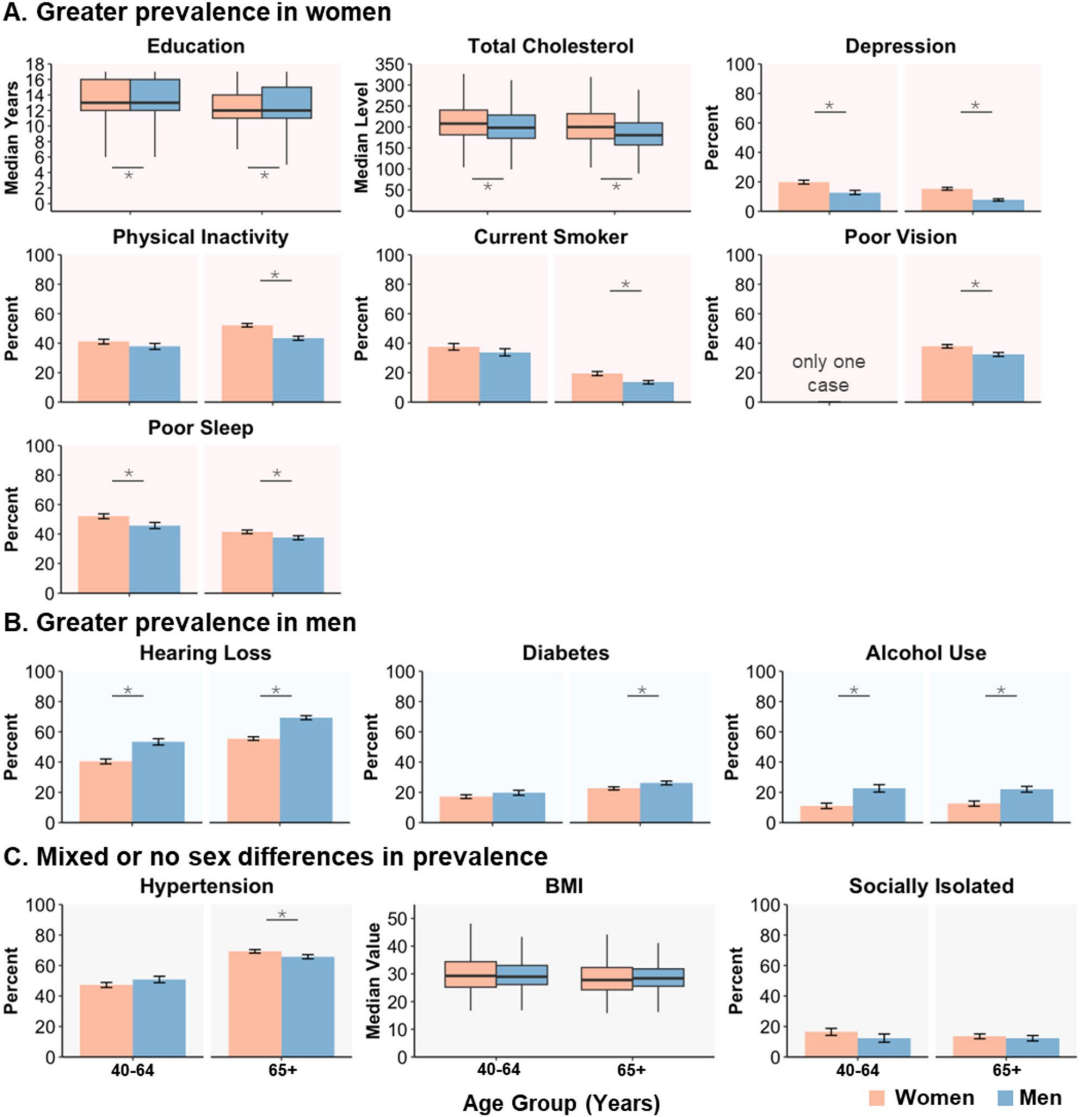
Sex and age differences in prevalence (or median) of 13 risk factors of dementia. Plots of prevalence (shown as percent) or median value (for continuous risk factors) separated by sex and age group. Panel A shows risk factors with a greater prevalence (or median) in women compared to men. Education is included in this section because the mean years in each age group are slightly lower in women compared to men. Panel B shows risk factors with a greater prevalence in men compared to women. Panel C shows risk factors with no significant overall sex differences in prevalence (or median) or with sex differences that vary by age group. Asterisk denotes significant (*p* <.05, FDR corrected) differences between men and women. Error bars on bar graphs show 95% confidence intervals. Note that for continuous risk factors, education, total cholesterol, and BMI, boxplots show median values while statistical tests were conducted on mean values

Complete statistics for comparisons in the prevalence (or mean) of each risk factor between women and men in the entire sample and within each age group are reported in Additional File 2. To summarize these findings (see Fig. 2), women exhibited greater prevalence of seven risk factors compared to men. In general, women had slightly fewer years of education, elevated total cholesterol, and a higher prevalence of depression, physical inactivity, smoking, poor vision, and poor sleep compared to men. Conversely, men had higher prevalence of three risk factors compared to women, including hearing loss, diabetes, and excessive alcohol use. One risk factor showed mixed sex differences, depending on the age group. The prevalence of hypertension was slightly higher in men between the ages of 40 to 64 (though not statistically significant after correction for multiple comparisons), but after age 65, the prevalence was significantly greater in women. Lastly, there were no sex differences in mean BMI nor in the prevalence of social isolation.

### Sex differences in associations between risk factors and cognitive performance

**Fig. 3:**
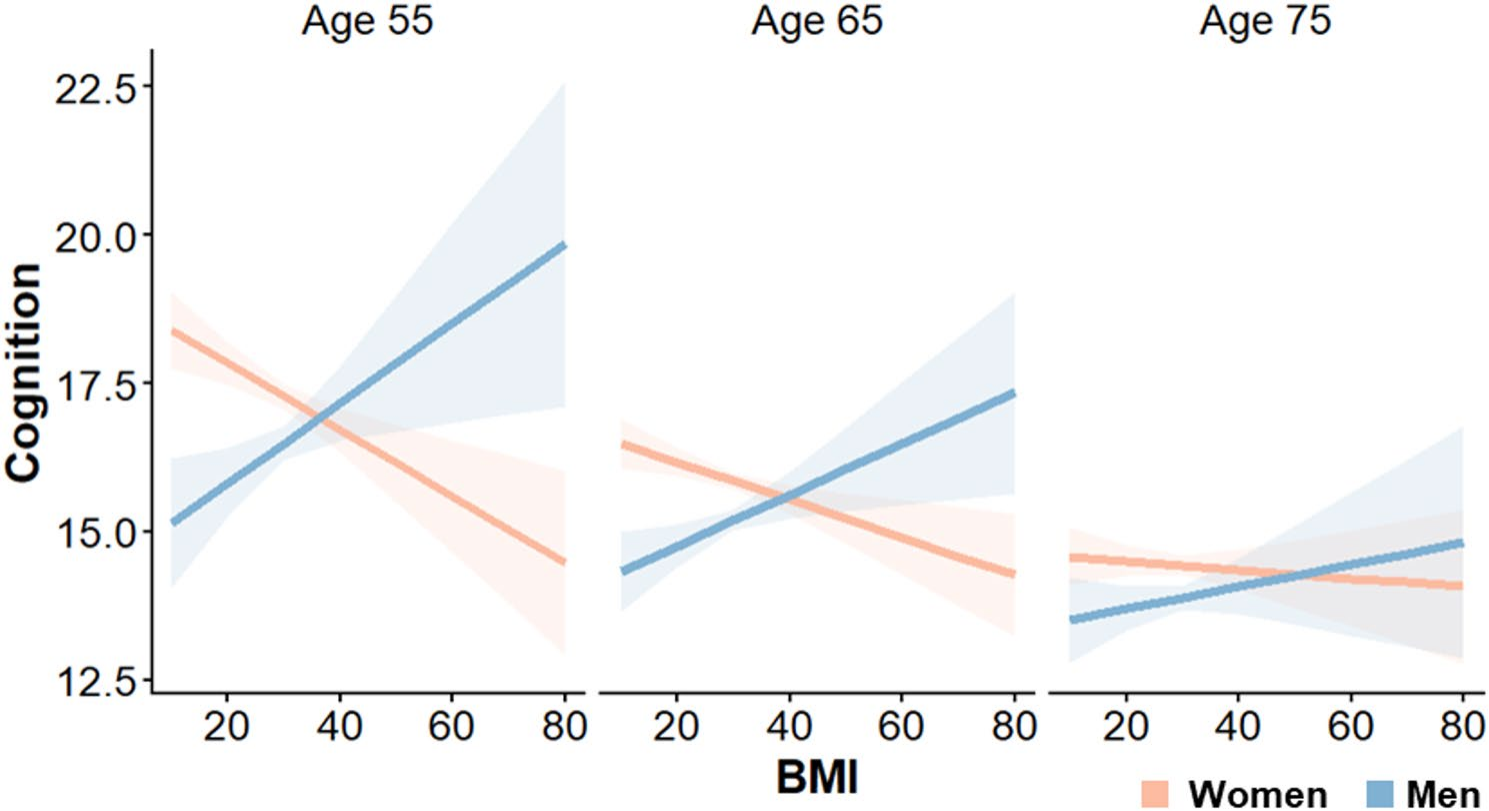
Sex and age differences in associations between BMI and cognitive performance. Plots of correlations between body mass index (BMI) and cognitive performance separated by sex and shown at three representative ages. In women, higher BMI is associated with poorer cognitive scores

Sex and age differences in the associations between each of the 13 risk factors and the cognitive summary score were examined. Complete model statistics of all three-way interactions of risk factor, sex, and age on cognition, and their simple effects, are reported in Additional File 2 Table 1). There was a significant three-way interaction between BMI, sex, and age (β = −0.005, SE = 0.002, *p* =.011). Regression coefficients represent the change in the cognitive summary score (range 0–27) associated with a one-unit increase in BMI. In women, there were negative associations between BMI and cognition at ages 55 and 65 (β = −0.05, SE = 0.02, *p* <.001 and β = −0.03, SE = 0.01, *p* =.002, respectively). Conversely, men showed positive associations between BMI and cognition (β = 0.07, SE = 0.03, *p* =.015 and β = 0.04, SE = 0.02, *p* =.012, respectively). Pairwise comparisons showed that these associations were significantly different between women and men (β = −0.12, SE = 0.03, *p* <.001 at age 55 and β = −0.08, SE = 0.02, *p* <.001 at age 65) (Fig. 3). However, the associations between BMI and cognition did not differ by sex at age 75 (β = −0.03, SE = 0.02, *p* =.256). No other risk factor showed significant three-way interactions.

**Fig. 4:**
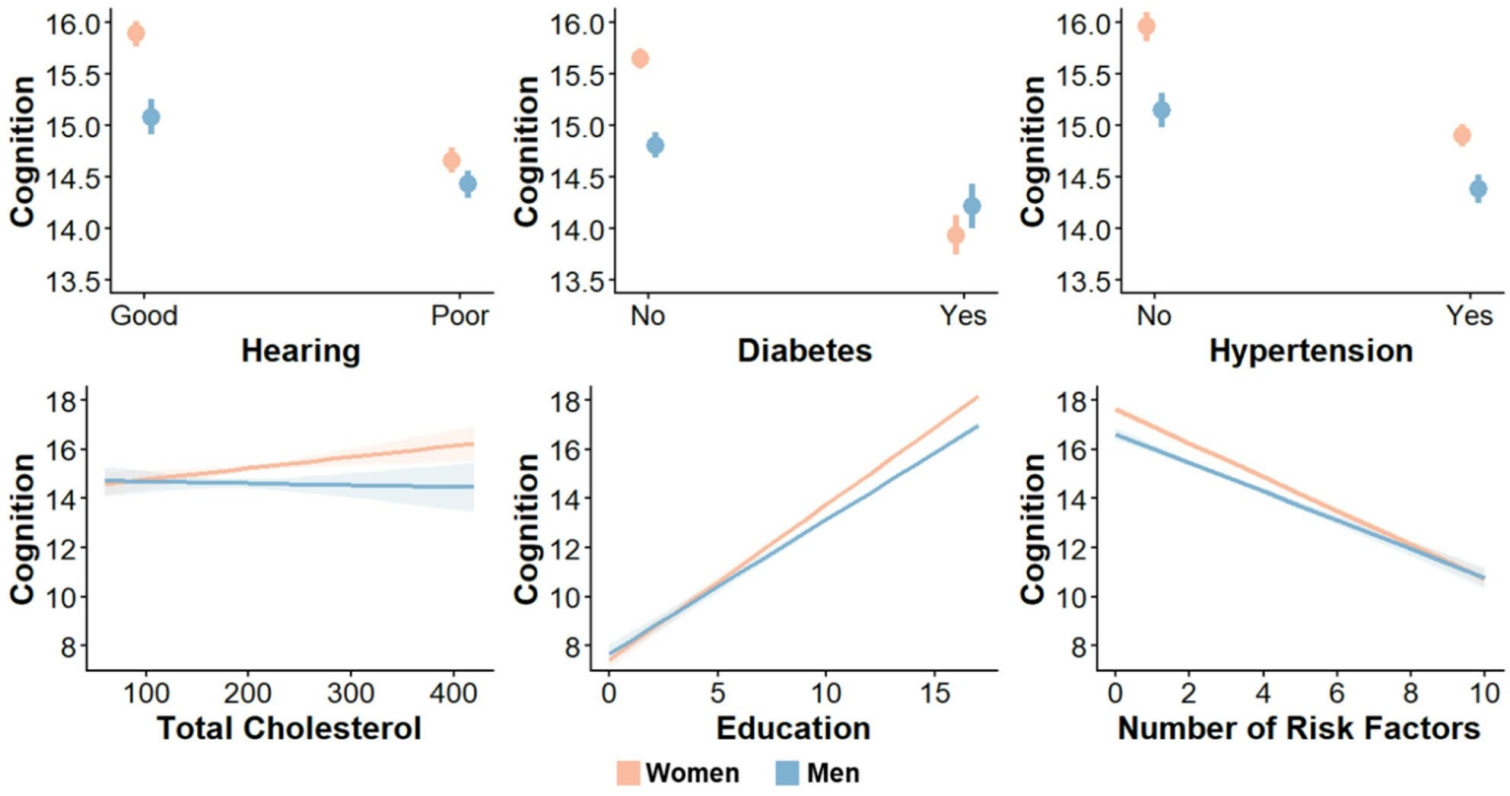
Sex differences in associations between risk factors of dementia and cognitive performance. Plots of mean cognitive performance associated with the presence or absence of risk factors (or correlations for continuous risk factors) separated by sex. Plots show that risk factors exert greater effects on cognition in women compared to men

In reduced models, several risk factors exhibited significant two-way interactions between risk factor and sex, with greater effects on cognition in women compared to men (Fig. 4, complete model statistics of two-way interactions and simple effects in Additional File 2, Table 2). Regression coefficients in these models represent the change in the cognitive summary score (range 0–27) in the group with the risk factor compared to the group without the risk factor, or per one-unit increase in continuous risk factors. The presence of hearing loss had a greater, negative effect on cognition in women (β = −1.23, SE = 0.09, *p* <.001) compared to men (β = −0.65, SE = 0.11, *p* <.001; women vs. men β = 0.57, SE = 0.14, *p* <.001). Diabetes also had a greater, negative effect on cognition in women (β = −1.71, SE = 0.11, *p* <.001) compared to men (β = − 0.59, SE = 0.13, *p* <.001; women vs. men β = 1.12, SE = 0.16, *p* <.001). Similarly, hypertension had a greater, negative effect on cognition in women (β = −1.06, SE = 0.09, *p* <.001) than men (β = −0.76, SE = 0.11, *p* <.001; women vs. men β = 0.29, SE = 0.14, *p* =.038). Conversely, there was a positive association between level of education and cognitive scores in both sexes (women: β = 0.63, SE = 0.01, *p* <.001; men: β = 0.55, SE = 0.01, *p* <.001), yet the association was stronger in women (women vs. men (the reference group) β = −0.08, SE = 0.02, *p* <.001). The association between total cholesterol and cognition also differed by sex (β = −0.01, SE < 0.01, *p* =.049), with women showing a significant, positive association (β = 0.01, SE < 0.01, *p* =.005) and men showing no significant association (β = < −0.01, SE < 0.01, *p* =.735).

While there was not a significant three-way interaction between the composite risk score, sex, and age on cognition, there was a significant two-way interaction between the composite risk score and sex on cognition (β = 0.11, SE = 0.04, *p* =.005; see Tables 1 and 2 in Additional File 2). Both women (β = −0.70, SE = 0.02, *p* <.001) and men (β = −0.59, SE = 0.03, *p* <.001) showed negative associations between number of risk factors and cognition, but the decrease in cognition with increasing number of risk factors was steeper in women (Fig. 4, last panel). There were no significant interactions between composite risk score adjusted for shared variance and sex on cognition (see Tables 1 and 2 in Additional File 2).

Secondary analyses were performed such that the three-way interactions of risk factor, age, and sex on the cognitive summary score, and the reduced models with two-way interactions of risk factor and sex, included education as a covariate. The results in these analyses followed the same trends as the primary analysis. Complete results are reported in Additional File 2 (Tables 3 and 4).

## Discussion

In a representative cohort of older adults in the United States, we examined 13 modifiable risk factors of dementia and identified sex differences in risk factor prevalence and in their associations with cognition. Contrary to our hypothesis, our findings showed that risk factor prevalence was greater among women compared to men. Women had higher rates of depression, physical inactivity, smoking, poor vision, and poor sleep compared to men, as well as lower levels of education and higher total cholesterol levels. Comparatively, men had higher rates of only three risk factors: hearing loss, diabetes, and excessive alcohol use. The associations between risk factors and cognitive performance differed by sex. Women generally had higher cognitive scores than men in the absence of a given risk factor, but the presence of risk factors was associated with greater reductions in cognitive scores in women than in men. Specifically, hearing loss, diabetes, hypertension, and BMI, had larger, negative effects on cognition in women compared to men. While two risk factors, years of education and total cholesterol, showed positive associations with cognition, such that higher levels were correlated with greater cognition. The accumulation of multiple risk factors was also associated with worse cognition in women compared to men. The absence of significant 3-way interactions for most risk factors suggests that sex differences in the association between these risk factors and cognition were relatively consistent across the age groups examined. Together, our study suggests that risk factors of dementia may influence cognition to a greater degree in women than in men.

As described earlier, the previous literature shows the prevalence of modifiable risk factors of dementia differ between women and men. The literature consistently reports that women are more likely to experience lower levels of education, high cholesterol, physical inactivity, and depression, while more men experience diabetes, hearing loss, and substance abuse [17–21]. Our study extends our understanding of sex differences to include a greater prevalence of poor visual health and poor self-reported sleep quality in women compared to men. Previous work further shows that many of these risk factors, like hypertension, increase dementia risk to a greater degree in women compared to men [18, 31], supporting our findings that the presence of risk factors reduce cognitive performance more in women than men.

Across the literature and within our study, hypertension is consistently associated with poorer cognitive outcomes in women compared to men. We found that hypertension had a stronger, negative association with cognitive performance in women. Several empirical studies and systematic reviews report that midlife hypertension is associated with greater cognitive decline and risk of dementia in women than men [32–36]. Recent comprehensive reviews suggests that women may be more prone to hypertension and other cardiovascular conditions due to experiencing pregnancy complications (i.e., gestational hypertension), hormonal shifts associated with menopause, and having a higher prevalence of cerebral small vessel disease, though the mechanisms linking these events with increased hypertension risk are not well-understood [37, 38].

Other co-morbidities of hypertension similarly affected women to a greater degree than men. Despite being more prevalent in men, diabetes was associated with a greater reduction in cognitive performance in women compared to men. Other recent longitudinal studies also report steeper declines in cognition over time in women compared to men with diabetes [39, 40]. Hearing loss was also associated with a greater reduction in cognition in women compared to men, despite being more prevalent in men. Conversely, mean BMI values were roughly equal between men and women, but exerted sex- and age-specific effects on cognition. Higher BMI values were associated with poorer cognitive performance in women at ages 55 and 65, yet had potentially positive effects on cognition in men.

The literature on BMI and cognition is mixed, which complicates interpretation of the sex differences observed in our results. Some studies suggest that higher BMI may have stronger adverse cognitive consequences for women than for men earlier in adulthood. For example, one study reported that higher BMI in midlife was associated with poorer cognitive performance in women but not men [41], and another found that elevated BMI earlier in life was associated with increased Alzheimer’s disease risk in women only [42]. One potential explanation is that obesity and related metabolic conditions may exert greater detrimental cognitive effects in women, particularly during midlife and early older adulthood when hormonal and metabolic changes associated with the menopausal transition occur. At the same time, the age-related pattern observed in our study is consistent with prior work describing the “obesity paradox,” whereby obesity in midlife is associated with increased dementia risk, while higher BMI in later life is associated with lower dementia risk [43, 44]. In this context, higher body weight in later older age may confer similar protective associations for both women and men. These findings highlight the need for further research on sex differences in vascular and metabolic risk factors, and their relationships with hormonal changes through the lifespan, in order to better mitigate the influence these risk factors have on cognition and dementia risk, particularly in women.

### Limitations

While the Health and Retirement Study collected a wide array of variables, many risk factors of interest could only be obtained from a single or small number of available variables. For example, there were only self-reported assessments of some health conditions and behaviors, like hearing loss and physical activity. Due to the scope of data collected by the Health and Retirement Study, the use of more extensive and validated questionnaires or clinical measures was likely not feasible. Future studies on risk factors should aim to use variables derived from validated questionnaires or health records, to supplement self-reported information from single items. The way in which some variables were operationalized, such as binarizing an ordinal response, loses more nuanced information available in the range of the response. However, the goal of this study was to examine prevalence and impact of the presence of several risk factors, therefore, binarizing risk factors into “yes, present” and “no, not present” was necessary. While the goal of this study was to assess a wide range of risk factors, future studies should also seek to examine these relationships controlling for additional, explanatory covariates, such as socioeconomic status. Lastly, it is possible that the sex differences observed in this study are at least partially explained by a survival bias in women, wherein men in poor health and with poorer cognitive performance withdraw from the study in greater numbers than women. However, the proportion of men to women in the sample remained consistent until ages 90 and older, suggesting that survival bias was less likely to play a major role in our findings.

### Perspectives and Significance

It is important to distinguish between sex differences in the prevalence of risk factors and their impact on cognition, because prevalence and impact may not correspond. Targeting only the most prevalent risk factors within each sex may overlook certain risk factors that more markedly influence cognitive decline. For instance, hearing loss is more prevalent in men but impacted cognition to a greater degree in women. Similarly, other work has shown that hearing loss in women is associated with a greater risk of dementia compared to men [45]. Risk reduction strategies of dementia should be sure to prioritize risk factors with the strongest association with cognitive outcomes in each sex, and not merely those that are most prevalent.

## Conclusions

In summary, our study suggests that women may be at greater risk of dementia because they experience a greater number of risk factors, and because these risk factors reduce cognition to a greater degree than men. These findings help to provide personalized, sex-based guidance for interventions on risk reduction. Our study suggests that women have a greater number of risk factors that can be targeted to mitigate their risk of dementia. Women particularly may want to seek treatments or management for hearing loss, bouts of insomnia, hypertension, diabetes, and excess weight, particularly in midlife and early older adulthood. Continued research is needed to fully understand sex differences in dementia so that we can develop better lifestyle intervention designs and reduce both the prevalence and impact of the disease.

## Supplementary Information

Supplementary Material 1

Supplementary Material 2

## Data Availability

The dataset supporting the conclusions of this article are available from the Heath and Retirement Study ([https://hrs.isr.umich.edu/](https:/hrs.isr.umich.edu)).
