# Supplementary material for: Sex differences in modifiable risk factors of dementia and their associations with cognition
Source: Biol Sex Differ. 2026 May 20;17:110. doi: 10.1186/s13293-026-00908-7 (PMC13188715; doi:10.1186/s13293-026-00908-7)
Supplement: Supplementary file 1 — Supplementary Material 1 [file 13293_2026_908_MOESM1_ESM.docx]

**Operationalization of risk factors**

For all risk factors, participants who responded “do not know” or refused to answer were excluded from the analyses.

Education was obtained from a question on the highest grade of school or year of college completed and was a continuous variable. A score of zero was assigned for no formal education. Scores 1 through 11 were assigned for completing grades 1 through 11. A score of 12 was assigned for obtaining a high school diploma. A score of 13-15 was assigned for completing some college. A score of 16 was assigned for obtaining a college degree. Lastly, a score of 17 was assigned for completing any amount of formal education beyond obtaining a college degree.

Hearing status was obtained from a question asking the participant to rate their perceived hearing. The response options included, “excellent,” “very good,” “good,” “fair,” and “poor.” Mirroring similar questionnaires on self-rated hearing ability, responses of “excellent” and “very good” were coded as good hearing, while responses of “good,” “fair,” and “poor” were coded as hearing loss [1,2]. Other work has shown that self-reported hearing ability has approximately 70% sensitivity compared to objective hearing tests [3].

Cholesterol levels were obtained from an analysis of dried blood spots, which were collected in the 2006-2016 study waves. Given the shift in the field away from dried blood spots to venous blood collection, and the difference in values between these methods, the Health and Retirement Study team previously transformed the dried blood spot values to be equivalent to venous blood values (further details regarding the rationale and methods can be reviewed here [4]). These values were made available to other users of the Health and Retirement Study data. Assays for total cholesterol and high-density lipoprotein cholesterol, but not low-density lipoprotein cholesterol were obtained. Our study uses the transformed total cholesterol values in mg/dL, as recommended by the Health and Retirement Study team [4].

Depression status was obtained from a question asking if the participant “felt sad, blue, or depressed” for two or more consecutive weeks in the prior 12 months. The response options included, “yes,” “no,” or “did not feel depressed because on anti-depressant medication.” Participants who responded that they did not experience depression due to taking medication were excluded from the analyses.

Physical inactivity was obtained from two questions on frequency of either moderate or vigorous activity. Moderate physical activity was described in the questionnaire as activities like “light gardening, cleaning the car, walking at a moderate pace, dancing, and floor or stretching exercises.” Vigorous physical activity was described as activities like “running or jogging, swimming, cycling, aerobics or gym workout, tennis, or digging with a spade or shovel.” For both moderate and vigorous physical activity, the responses for activity frequency included, “every day,” “more than once a week,” “once a week,” “one to three times a month,” and “hardly ever or never.” Physical inactivity was coded as responding to both vigorous and moderate physical activity with once a week or less frequency.

Diabetes status was obtained from a question asking the participant if a doctor previously diagnosed them with diabetes or high blood sugar. If the participant was newly enrolled in this wave, the response options were, “yes” or “no.” If the participant had completed previous waves, they were asked to confirm their previous endorsement of their diabetes status. The response options for these participants included, “yes,” “no,” “disputes previous wave record, but now has condition,” and “disputes previous wave record, does not have condition.” Those who disputed their previous response were excluded from the analyses.

Smoking status was obtained from a question asking the participant if they smoke cigarettes currently. The response options included, “yes” or “no.”

Hypertension status was obtained from a question asking the participant if a doctor previously diagnosed them with high blood pressure or hypertension. If the participant was newly enrolled in this wave, the response options were, “yes” or “no.” If the participant had completed previous waves, they were asked to confirm their previous endorsement of their hypertension diagnosis. The response options for these participants included, “yes,” “no,” “disputes previous wave record, but now has condition,” and “disputes previous wave record, does not have condition.” Those who disputed their previous response were excluded from the analyses.

Obesity was obtained by calculating the body mass index (BMI) for each participant using measurements of height (in inches) and weight (in pounds) obtained during the physical assessment. BMI was calculated as a continuous variable using the standard equation for imperial units, $\left( \frac{weight}{{height}^{2}} \right)*703$. Histograms were examined to identify extreme outlying values (presumably, data entry errors) in both height and weight measurements. Participants with values less than 30 inches for height and less than 60 pounds for weight were excluded from the analyses (n=9).

Alcohol use was obtained by calculating the number of alcoholic drinks per week based on responses to two questions. The number of days per week the participant drinks was multiplied by the number of drinks per day the participant consumes. Participants who consumed 13 or more drinks per week were coded as having excessive alcohol consumption, as defined by the Lancet Commission [5].

Social isolation was obtained by summarizing and normalizing seven items derived from the Psychological and Lifestyle Questionnaire, following previously published methods [6,7]. Briefly, the seven items included: 1) network size, which was computed as the total number of family and friends with which the participant has a close relationship; 2) network range, which was computed as the number of close relationship types (children, family, or friends); 3) number of friends with which the participant has a close relationship; 4) the frequency with which the participant has any contact with the people in their network; and 5) how often the participant meets in person with their children; 6) family; and 7) friends. For items four through seven, responses and corresponding point values included, “1=less than once a year,” “2=once or twice a year,” “3=every few months,” “4=once or twice a month,” “5=once or twice a week,” “6=three or more times a week.” These seven items were summed and z-scored. Those who were greater than one standard deviation below the mean were considered socially isolated. Those who were within one standard deviation or greater than one standard deviation above the mean were considered not isolated.

Poor vision was obtained from questions asking the participant if they previously had been treated for glaucoma or had undergone cataract surgery. The response options included, “yes” or “no.” A “yes” response to either question was coded as having poor vision.

Poor sleep was obtained from a question asking the participant if they feel rested upon waking in the morning. Response options included, “most of the time,” “sometimes,” “rarely or never.” Those who responded with “sometimes” or “rarely or never” were categorized as having poor sleep.

**References:**

[1] Kamil RJ, Genther DJ, Lin FR. Factors Associated With the Accuracy of Subjective Assessments of Hearing Impairment. Ear and Hearing 2015;36:164. https://doi.org/10.1097/AUD.0000000000000075.

[2] Tsimpida D, Kontopantelis E, Ashcroft D, Panagioti M. Comparison of Self-reported Measures of Hearing With an Objective Audiometric Measure in Adults in the English Longitudinal Study of Ageing. JAMA Netw Open 2020;3:e2015009. https://doi.org/10.1001/jamanetworkopen.2020.15009.

[3] Kim AS, Betz JF, Albert M, Deal JA, Faucette SP, Oh ES, et al. Accuracy of self- and proxy-rated hearing among older adults with and without cognitive impairment. Journal of the American Geriatrics Society 2022;70:490–500. https://doi.org/10.1111/jgs.17558.

[4] Crimmins E, Faul J, Kim J, Guyer H, Langa K, Ofstedal M, et al. Documentation of Biomarkers in the 2006 and 2008 Health and Retirement Study 2013.

[5] Livingston G, Huntley J, Liu KY, Costafreda SG, Selbæk G, Alladi S, et al. Dementia prevention, intervention, and care: 2024 report of the Lancet standing Commission. The Lancet 2024;404:572–628. https://doi.org/10.1016/S0140-6736(24)01296-0.

[6] Cornwell EY, Waite LJ. Social Disconnectedness, Perceived Isolation, and Health among Older Adults. J Health Soc Behav 2009;50:31–48. https://doi.org/10.1177/002214650905000103.

[7] Shaw JG, Farid M, Noel-Miller C, Joseph N, Houser A, Asch SM, et al. Social Isolation and Medicare Spending: Among Older Adults, Objective Isolation Increases Expenditures While Loneliness Does Not. J Aging Health 2017;29:1119–43. https://doi.org/10.1177/0898264317703559.
