# Supplementary material for: Sex differences in modifiable risk factors of dementia and their associations with cognition
Source: Biol Sex Differ. 2026 May 20;17:110. doi: 10.1186/s13293-026-00908-7 (PMC13188715; doi:10.1186/s13293-026-00908-7)
Supplement: Supplementary file 2 — Supplementary Material 2 [file 13293_2026_908_MOESM2_ESM.docx]

**Differences in risk factor prevalence (or mean) by sex**

Chi-square tests were used to assess sex differences in the prevalence of dichotomous dementia risk factors, while *t*-tests were used to assess the mean difference in continuous risk factors (education, total cholesterol, and BMI). Sex differences were assessed between all men and women available in the sample, and between men and women within age groups of 40-64 and 65 and older, for each risk factor. Below, we report the prevalence or mean for each risk factor and the associated test statistic. The p-values are shown unadjusted, but we indicate with a * which results survive correction for multiple comparisons using the Bonferroni method (13 risk factors * 3 tests = 39 separate tests, adjusted alpha 0.05/39=0.001). Given the large sample size, we also chose to include effect sizes so that the reader may interpret the magnitude of differences with their own criteria.

*Education*

Within the entire sample, the mean(sd) level of education for women was 12.33 (3.12) years and 12.61 (3.45) years for men (*t*(13866.19)=-5.44, p<.001*, Cohen’s *d*=0.09, (Levene’s test for equality of variances was significant (F=144.82, p<.001)). Within the 40-64 years age group, the mean (sd) education for women was 12.99 (2.98) and 13.30 (3.07) for men (*t*(4539.97)=-3.69, p<.001*, *d*=.10, (Levene’s test for equality of variances was significant (F=10.48, p=.001)). Within the 65 years and older age group, the mean (sd) education for women was 11.97 (3.14) and 12.29 (3.57) for men (*t*(9363.00)=-4.95, p<.001*, *d*=.10 (Levene’s test for equality of variances was significant (F=154.28, p<.001)).

*Hearing Loss*

Within the entire sample, the prevalence of self-reported poor hearing differed by sex, with 50.2% in women and 64.2% in men (X^2^(1)=330.90, p<.001*, φ=0.14). Within the 40-64 years age group, 40.4% of women and 53.4% of men reported poor hearing (X^2^(1)=94.44, p<.001*, φ=0.13). In the 65 years and older age group, 55.6% of women and 69.4% of men reported poor hearing (X^2^(1)=221.19, p<.001*, φ=0.14).

*Total Cholesterol*

Within the entire sample, the mean (sd) levels of total cholesterol were significantly higher for women at 207.12 (42.55) mg/dL compared to 191.66 (39.28) mg/dL for men (*t*(5395.49)=14.45, p<0.001*, Cohen’s *d*=0.38 (Levene’s test for equality of variances was significant (F=20.72, p<.001)). Within the 40-64 years age group, the mean (sd) total cholesterol level for women was 211.89 (42.45) and 202.87 (40.77) for men (*t*(2061)=4.70, p<.001*, *d*=.22). Within the 65 years and older age group, the mean (sd) total cholesterol level for women was 204.41 (42.37) and 186.59 (37.52) for men (*t*(3774.63)=13.95, p<.001*, Cohen’s *d*=0.44 (Levene’s test for equality of variances was significant (F=26.18, p<.001)).

*Depression*

Within the entire sample, the prevalence of feeling depressed differed by sex, with 17.0% of women and 9.4% of men endorsing depression (X^2^(1)=184.94, p<.001*, φ=0.11). Within the 40-64 years age group, 19.8% of women and 12.8% of men endorsed depression (X^2^(1)=45.58, p<.001*, φ=0.09). In the 65 years and older age group, 15.3% of women and 7.7% of men endorsed depression (X^2^(1)=137.22, p<.001*, φ=0.12).

*Physical Inactivity*

Within the entire sample, the prevalence of physical inactivity differed by sex, with 48.2% of women and 41.6% of men reporting that they do not engage in frequent physical activity (X^2^(1)=74.77, p<.001*, φ=0.07). Within the 40-64 years age group, 41.0% of women and 37.8% of men were physically inactive (X^2^(1)=5.84, p=.016, φ=0.03). In the 65 years and older age group, 52.2% of women and 43.3% of men were inactive (X^2^(1)=86.94, p<.001*, φ=0.09).

*Diabetes*

Within the entire sample, the prevalence of diabetes differed by sex, with 20.7% of women and 24.1% of men reporting that they had diabetes (X^2^(1)=28.19, p<.001*, φ=0.04). Within the 40-64 years age group, 17.2% of women and 19.7% of men had diabetes (X^2^(1)=5.95, p=.015, φ=0.03). In the 65 years and older age group, 22.6% of women and 26.2% of men had diabetes (X^2^(1)=19.40, p<.001*, φ=0.04).

*Current Smoker*

Within the entire sample, the prevalence of individuals who currently smoked differed by sex, with 26.1% of women and 19.7% of men reporting that they smoked (X^2^(1)=56.57, p<.001*, φ=0.07). Within the 40-64 years age group, 37.5% of women and 33.8% of men reported smoking (X^2^(1)=4.90, p=.027, φ=0.04). In the 65 years and older age group, 19.4% of women and 13.5% of men smoked (X^2^(1)=40.95, p<.001*, φ=0.08).

*Hypertension*

Within the entire sample, the prevalence of hypertension did not differ between women (61.5%) and men (61.0%) (X^2^(1)=00.43, p=.511, φ=0.01). Within the 40-64 years age group 47.3% of women and 50.9% of men (X^2^(1)=7.09, p=.008, φ=0.04) reported hypertension. In the 65 years and older age group, 69.3% of women and 65.8% of men reported hypertension (X^2^(1)=15.43, p<.001*, φ=0.04).

*Body Mass Index (BMI)*

Within the entire sample, the mean (sd) BMI for women was 29.37 (6.66) and 29.20 (5.09) for men, which did not significantly differ (*t*(6334.09)=1.21, p=.226, Cohen’s *d*=0.03 (Levene’s test for equality of variances was significant (F=154.86, p<.001)). Within the 40-64 years age group, the mean (sd) BMI for women was 30.31 (6.87) and 29.73 (5.26) for men (*t*(2056.20)=2.21, p=.027, *d*=.09 (Levene’s test for equality of variances was significant (F=60.28, p<.001)). Within the 65 years and older age group, the mean (sd) BMI for women was 28.84 (6.47) and 28.95 (4.98) for men (*t*(4200.41)=-0.65, p=.519, *d*=.02 (Levene’s test for equality of variances was significant (F=84.45, p<.001)).

*Alcohol Use*

Within the entire sample, the prevalence of alcohol use greater than 12 servings per week differed by sex, with 11.9% of women and 22.3% of men reporting that they drank in excess (X^2^(1)=104.77, p<.001*, φ=0.14). Within the 40-64 years age group, 11.1% of women and 22.6% of men reported drinking in excess X^2^(1)=55.88, p<.001*, φ=0.16). In the 65 years and older age group, 12.5% of women and 22.0% of men reported drinking in excess (X^2^(1)=49.51, p<.001*, φ=0.12).

*Social Isolation*

Within the entire sample, the prevalence of social isolation was 14.6% of women and 12.3% of men (X^2^(1)=5.00, p=.025, φ=0.03). Within the 40-64 years age group, 16.5% of women and 12.4% of men felt socially isolated (X^2^(1)=4.77, p=.029, φ=0.06). Within the 65 years and older age group, 13.6% of women and 12.3% of men felt isolated (X^2^(1)=1.06, p=.304, φ=0.02).

*Poor Vision*

Within the entire sample, the prevalence of poor vision (i.e., glaucoma or cataracts) was 29.6% of women and 27.6% of men, X^2^(1)=6.78, p=.009, φ=0.02). There was only one case of poor vision in the 40-64 years age group (0.1% of women, 0.0% of men, X^2^(1)=0.45, p<.504, φ=0.01) Within the 65 years and older age group, 37.9% of women, 32.3% of men reported poor vision X^2^(1)=37.09, p<.001*, φ=0.06.

*Poor Sleep*

Within the entire sample, the prevalence of poor sleep differed by sex, with 45.2% of women and 40.1% of men reporting experiencing poor sleep (X^2^(1)=44.10, p<.001*, φ=0.05). The prevalence of poor sleep differed by sex within the 40-64 years age group (52.0% of women, 45.7% of men, X^2^(1)=21.99, p<.001*, φ=0.06) and the 65 years and older age group, with 41.5% of women and 37.4% of men reporting poor sleep (X^2^(1)=18.73, p<.001*, φ=0.04).

**Table 1. Three-way interactions between risk factor, sex, and age on cognition for the 13 risk factors**

Each three-way interaction was probed at three representative ages (55, 65, and 75), testing two-way interactions between risk factor and sex on cognition. Age and age^2^ were mean centered. The p-values are shown unadjusted, but we indicate with a * which simple effect results survive correction for multiple comparisons using the Bonferroni method (6 separate tests for dichotomous risk factors, adjusted alpha 0.05/6=0.008; 3 separate tests for continuous risk factors, adjusted alpha 0.05/3=0.016). Betas are unstandardized.

| **Model Terms** | **Estimate (β)** | **Standard Error** | **95% CI**  **lower** | **95% CI**  **upper** | **t-value / t-ratio** | **p-value** |
| --- | --- | --- | --- | --- | --- | --- |
| **Education** |  |  |  |  |  |  |
| Intercept | 7.841 | 0.168 | 7.511 | 8.171 | 46.591 | <.001 |
| Education | 0.627 | 0.013 | 0.602 | 0.653 | 48.096 | <.001 |
| Sex | 0.066 | 0.255 | -0.434 | 0.566 | 0.260 | 0.795 |
| Age | -0.113 | 0.015 | -0.143 | -0.084 | -7.49 | <.001 |
| Age^2^ | -0.004 | <.001 | -0.004 | -0.003 | -16.167 | <.001 |
| 2‑way: Education * Sex | -0.072 | 0.020 | -0.110 | -0.033 | -3.655 | <.001 |
| 2‑way: Education * Age | -0.001 | 0.001 | -0.004 | 0.001 | -1.059 | 0.290 |
| 2‑way: Sex * Age | 0.048 | 0.024 | 0.000 | 0.096 | 1.962 | 0.050 |
| 3-way: Education * Sex * Age | -0.002 | 0.002 | -0.006 | 0.001 | -1.300 | 0.194 |
| Simple Effect: Δ Slopes F – M @ 55 yrs | 0.037 | 0.034 | -0.030 | 0.104 | 1.081 | 0.280 |
| Simple Effect: Δ Slopes F – M @ 65 yrs | 0.061 | 0.022 | 0.019 | 0.104 | 2.830 | 0.005 |
| Simple Effect: Δ Slopes F – M @ 75 yrs | 0.086 | 0.022 | 0.043 | 0.128 | 3.952 | <.001 |
| **Poor Hearing** |  |  |  |  |  |  |
| Intercept | 16.176 | 0.068 | 16.042 | 16.309 | 237.503 | <.001 |
| Hearing | -1.170 | 0.088 | -1.343 | -0.997 | -13.223 | <.001 |
| Sex | -0.767 | 0.110 | -0.982 | -0.551 | -6.959 | <.001 |
| Age | -0.167 | 0.006 | -0.179 | -0.155 | -27.624 | <.001 |
| Age^2^ | -0.004 | <.001 | -0.004 | -0.003 | -14.958 | <.001 |
| 2‑way: Hearing * Sex | 0.523 | 0.143 | 0.242 | 0.803 | 3.655 | <.001 |
| 2‑way: Hearing * Age | 0.042 | 0.008 | 0.026 | 0.058 | 5.015 | <.001 |
| 2‑way: Sex * Age | 0.015 | 0.011 | -0.007 | 0.037 | 1.359 | 0.174 |
| 3-way: Hearing * Sex * Age | -0.011 | 0.014 | -0.039 | 0.017 | -0.775 | 0.438 |
| Simple Effect: Poor – Good, F @ 55 | -1.767 | 0.142 | -2.046 | -1.488 | -12.416 | <0.001 |
| Simple Effect: Poor – Good, M @ 55 | -1.088 | 0.189 | -1.459 | -0.718 | -5.754 | <0.001 |
| Simple Effect: Poor – Good, F @ 65 | -1.347 | 0.093 | -1.529 | -1.166 | -14.56 | <0.001 |
| Simple Effect: Poor – Good, M @ 65 | -0.778 | 0.118 | -1.009 | -0.548 | -6.617 | <0.001 |
| Simple Effect: Poor – Good, F @ 75 | -0.928 | 0.104 | -1.132 | -0.724 | -8.907 | <0.001 |
| Simple Effect: Poor – Good, M @ 75 | -0.469 | 0.137 | -0.737 | -0.200 | -3.421 | 0.001 |
| **Total Cholesterol** |  |  |  |  |  |  |
| Intercept | 14.603 | 0.349 | 13.918 | 15.287 | 41.838 | <0.001 |
| Cholesterol | 0.004 | 0.002 | 0.001 | 0.008 | 2.738 | 0.006 |
| Sex | 0.411 | 0.553 | -0.672 | 1.495 | 0.744 | 0.457 |
| Age | -0.129 | 0.033 | -0.194 | -0.064 | -3.890 | <0.001 |
| Age^2^ | -0.003 | <0.001 | -0.004 | -0.002 | -7.800 | <0.001 |
| 2‑way: Cholesterol * Sex | -0.005 | 0.003 | -0.010 | 0.001 | -1.853 | 0.064 |
| 2‑way: Cholesterol * Age | <0.001 | <0.001 | <-0.001 | 0.001 | -0.523 | 0.601 |
| 2‑way: Sex * Age | -0.033 | 0.055 | -0.141 | 0.075 | -0.596 | 0.551 |
| 3-way: Cholesterol * Sex * Age | <0.001 | <0.001 | <-0.001 | 0.001 | 0.630 | 0.529 |
| Simple Effect: Δ Slopes F – M @ 55 yrs | 0.008 | 0.005 | -0.001 | 0.017 | 1.641 | 0.101 |
| Simple Effect: Δ Slopes F – M @ 65 yrs | 0.006 | 0.003 | <0.001 | 0.012 | 2.010 | 0.044 |
| Simple Effect: Δ Slopes F – M @ 75 yrs | 0.004 | 0.003 | -0.002 | 0.011 | 1.246 | 0.213 |
| **Depression** |  |  |  |  |  |  |
| Intercept | 15.765 | 0.056 | 15.655 | 15.875 | 280.959 | <0.001 |
| Depression | -0.878 | 0.118 | -1.110 | -0.647 | -7.435 | <0.001 |
| Sex | -0.657 | 0.074 | -0.801 | -0.512 | -8.909 | <0.001 |
| Age | -0.161 | 0.004 | -0.17 | -0.152 | -36.397 | <0.001 |
| Age^2^ | -0.004 | <0.001 | -0.004 | -0.003 | -14.007 | <0.001 |
| 2‑way: Depression * Sex | -0.207 | 0.220 | -0.638 | 0.225 | -0.939 | 0.348 |
| 2‑way: Depression * Age | 0.025 | 0.011 | 0.004 | 0.047 | 2.321 | 0.020 |
| 2‑way: Sex * Age | 0.018 | 0.007 | 0.003 | 0.032 | 2.430 | 0.015 |
| 3-way: Depression * Sex * Age | 0.001 | 0.021 | -0.040 | 0.041 | 0.036 | 0.971 |
| Simple Effect: Yes – No, F @ 55 | -1.239 | 0.177 | -1.587 | -0.891 | -6.987 | <0.001 |
| Simple Effect: Yes – No, M @ 55 | -1.456 | 0.284 | -2.013 | -0.899 | -5.123 | <0.001 |
| Simple Effect: Yes – No, F @ 65 | -0.986 | 0.119 | -1.218 | -0.753 | -8.297 | <0.001 |
| Simple Effect: Yes – No, M @ 65 | -1.195 | 0.188 | -1.564 | -0.827 | -6.356 | <0.001 |
| Simple Effect: Yes – No, F @ 75 | -0.732 | 0.144 | -1.014 | -0.451 | -5.098 | <0.001 |
| Simple Effect: Yes – No, M @ 75 | -0.934 | 0.225 | -1.376 | -0.493 | -4.146 | <0.001 |
| **Physical Inactivity** |  |  |  |  |  |  |
| Intercept | 14.881 | 0.071 | 14.743 | 15.019 | 210.963 | <0.001 |
| Physical Inactivity | 1.316 | 0.088 | 1.144 | 1.487 | 15.036 | <0.001 |
| Sex | -0.739 | 0.105 | -0.945 | -0.534 | -7.044 | <0.001 |
| Age | -0.152 | 0.006 | -0.163 | -0.141 | -26.57 | <0.001 |
| Age^2^ | -0.003 | <0.001 | -0.004 | -0.003 | -12.900 | <0.001 |
| 2‑way: Physical Inactivity * Sex | 0.072 | 0.139 | -0.201 | 0.344 | 0.514 | 0.607 |
| 2‑way: Physical Inactivity * Age | 0.011 | 0.008 | -0.005 | 0.027 | 1.333 | 0.183 |
| 2‑way: Sex * Age | 0.020 | 0.010 | <0.001 | 0.039 | 1.985 | 0.047 |
| 3-way: Physical Inactivity * Sex * Age | -0.010 | 0.014 | -0.036 | 0.017 | -0.718 | 0.473 |
| Simple Effect: Yes – No, F @ 55 | -1.160 | 0.142 | -1.437 | -0.882 | 8.194 | <0.001 |
| Simple Effect: Yes – No, M @ 55 | -1.370 | 0.191 | -1.744 | -0.996 | 7.182 | <0.001 |
| Simple Effect: Yes – No, F @ 65 | -1.269 | 0.092 | -1.45 | -1.089 | 13.785 | <0.001 |
| Simple Effect: Yes – No, M @ 65 | -1.382 | 0.118 | -1.614 | -1.15 | 11.677 | <0.001 |
| Simple Effect: Yes – No, F @ 75 | -1.379 | 0.102 | -1.579 | -1.179 | 13.501 | <0.001 |
| Simple Effect: Yes – No, M @ 75 | -1.394 | 0.124 | -1.637 | -1.151 | 11.243 | <0.001 |
| **Current Smoker** |  |  |  |  |  |  |
| Intercept | 15.753 | 0.082 | 15.593 | 15.912 | 193.181 | <0.001 |
| Smoker | -0.935 | 0.158 | -1.245 | -0.626 | -5.927 | <0.001 |
| Sex | -0.755 | 0.102 | -0.955 | -0.555 | -7.396 | <0.001 |
| Age | -0.178 | 0.007 | -0.192 | -0.164 | -25.098 | <0.001 |
| Age^2^ | -0.003 | <0.001 | -0.004 | -0.002 | -8.407 | <0.001 |
| 2‑way: Smoker * Sex | -0.273 | 0.241 | -0.744 | 0.199 | -1.133 | 0.257 |
| 2‑way: Smoker * Age | 0.058 | 0.015 | 0.028 | 0.088 | 3.830 | <0.001 |
| 2‑way: Sex * Age | 0.027 | 0.010 | 0.007 | 0.047 | 2.591 | 0.010 |
| 3-way: Smoker * Sex * Age | -0.032 | 0.023 | -0.078 | 0.013 | -1.385 | 0.166 |
| Simple Effect: Yes – No, F @ 55 | -1.761 | 0.209 | -2.170 | -1.352 | -8.443 | <0.001 |
| Simple Effect: Yes – No, M @ 55 | -1.572 | 0.254 | -2.070 | -1.074 | -6.188 | <0.001 |
| Simple Effect: Yes – No, F @ 65 | -1.181 | 0.144 | -1.463 | -0.899 | -8.202 | <0.001 |
| Simple Effect: Yes – No, M @ 65 | -1.316 | 0.166 | -1.642 | -0.991 | -7.929 | <0.001 |
| Simple Effect: Yes – No, F @ 75 | -0.601 | 0.209 | -1.011 | -0.190 | -2.870 | 0.004 |
| Simple Effect: Yes – No, M @ 75 | -1.060 | 0.248 | -1.547 | -0.574 | -4.272 | <0.001 |
| **Diabetes** |  |  |  |  |  |  |
| Intercept | 16.01 | 0.058 | 15.896 | 16.123 | 276.277 | <0.001 |
| Diabetes | -1.703 | 0.108 | -1.914 | -1.492 | -15.821 | <0.001 |
| Sex | -0.829 | 0.078 | -0.982 | -0.675 | -10.584 | <0.001 |
| Age | -0.154 | 0.004 | -0.163 | -0.145 | -35.028 | <0.001 |
| Age^2^ | -0.004 | <0.001 | -0.004 | -0.003 | -15.888 | <0.001 |
| 2‑way: Diabetes * Sex | 1.093 | 0.165 | 0.771 | 1.416 | 6.646 | <0.001 |
| 2‑way: Diabetes * Age | 0.007 | 0.011 | -0.015 | 0.028 | 0.595 | 0.552 |
| 2‑way: Sex * Age | 0.016 | 0.007 | 0.001 | 0.031 | 2.143 | 0.032 |
| 3-way: Diabetes * Sex * Age | 0.007 | 0.017 | -0.027 | 0.041 | 0.382 | 0.702 |
| Simple Effect: Yes – No, F @ 55 | -1.239 | 0.177 | -2.165 | -1.425 | -6.987 | <0.001 |
| Simple Effect: Yes – No, M @ 55 | -1.456 | 0.284 | -1.257 | -0.336 | -5.123 | <0.001 |
| Simple Effect: Yes – No, F @ 65 | -0.986 | 0.119 | -1.959 | -1.501 | -8.297 | <0.001 |
| Simple Effect: Yes – No, M @ 65 | -1.195 | 0.188 | -0.940 | -0.390 | -6.356 | <0.001 |
| Simple Effect: Yes – No, F @ 75 | -0.732 | 0.144 | -1.911 | -1.420 | -5.098 | <0.001 |
| Simple Effect: Yes – No, M @ 75 | -0.934 | 0.225 | -0.814 | -0.252 | -4.146 | <0.001 |
| **Hypertension** |  |  |  |  |  |  |
| Intercept | 16.285 | 0.080 | 16.128 | 16.443 | 202.678 | <0.001 |
| Hypertension | -1.008 | 0.093 | -1.190 | -0.825 | -10.825 | <0.001 |
| Sex | -0.793 | 0.114 | -1.017 | -0.57 | -6.966 | <0.001 |
| Age | -0.154 | 0.007 | -0.166 | -0.141 | -23.521 | <0.001 |
| Age^2^ | -0.004 | <0.001 | -0.005 | -0.004 | -15.779 | <0.001 |
| 2‑way: Hypertension * Sex | 0.249 | 0.144 | -0.034 | 0.532 | 1.725 | 0.085 |
| 2‑way: Hypertension * Age | 0.018 | 0.009 | 0.001 | 0.034 | 2.038 | 0.042 |
| 2‑way: Sex * Age | -0.003 | 0.011 | -0.023 | 0.018 | -0.241 | 0.809 |
| 3-way: Hypertension * Sex * Age | 0.026 | 0.014 | -0.001 | 0.053 | 1.863 | 0.062 |
| Simple Effect: Yes – No, F @ 55 | -1.258 | 0.143 | -1.538 | -0.978 | -8.806 | <0.001 |
| Simple Effect: Yes – No, M @ 55 | -1.380 | 0.188 | -1.748 | -1.011 | -7.33 | <0.001 |
| Simple Effect: Yes – No, F @ 65 | -1.082 | 0.095 | -1.268 | -0.896 | -11.395 | <0.001 |
| Simple Effect: Yes – No, M @ 65 | -0.943 | 0.118 | -1.174 | -0.713 | -8.013 | <0.001 |
| Simple Effect: Yes – No, F @ 75 | -0.907 | 0.112 | -1.126 | -0.688 | -8.12 | <0.001 |
| Simple Effect: Yes – No, M @ 75 | -0.507 | 0.131 | -0.764 | -0.250 | -3.873 | <0.001 |
| **BMI** |  |  |  |  |  |  |
| Intercept | 16.32 | 0.315 | 15.702 | 16.938 | 51.735 | <0.001 |
| BMI | -0.023 | 0.010 | -0.043 | -0.002 | -2.195 | 0.028 |
| Sex | -2.32 | 0.566 | -3.429 | -1.211 | -4.101 | <0.001 |
| Age | -0.215 | 0.028 | -0.271 | -0.160 | -7.567 | <0.001 |
| Age^2^ | -0.003 | <0.001 | -0.004 | -0.002 | -7.498 | <0.001 |
| 2‑way: BMI * Sex | 0.057 | 0.019 | 0.019 | 0.094 | 2.975 | 0.003 |
| 2‑way: BMI * Age | 0.002 | 0.001 | 0.001 | 0.004 | 2.494 | 0.013 |
| 2‑way: Sex * Age | 0.158 | 0.056 | 0.049 | 0.267 | 2.833 | 0.005 |
| 3-way: BMI * Sex * Age | -0.005 | 0.002 | -0.009 | -0.001 | -2.546 | 0.011* |
| Simple Effect: Δ Slopes F – M @ 55 yrs | -0.123 | 0.032 | -0.186 | -0.061 | -3.901 | <0.001* |
| Simple Effect: Δ Slopes F – M @ 65 yrs | -0.075 | 0.020 | -0.114 | -0.036 | -3.743 | <0.001* |
| Simple Effect: Δ Slopes F – M @ 75 yrs | -0.026 | 0.023 | -0.071 | 0.019 | -1.136 | 0.256 |
| **Alcohol Use** |  |  |  |  |  |  |
| Intercept | 16.863 | 0.097 | 16.672 | 17.054 | 173.239 | <0.001 |
| Alcohol Use | -0.015 | 0.247 | -0.498 | 0.469 | -0.060 | 0.952 |
| Sex | -0.981 | 0.120 | -1.217 | -0.746 | -8.162 | <0.001 |
| Age | -0.144 | 0.008 | -0.159 | -0.128 | -18.088 | <0.001 |
| Age^2^ | -0.004 | <0.001 | -0.005 | -0.003 | -9.370 | <0.001 |
| 2‑way: Alcohol Use * Sex | -0.455 | 0.307 | -1.057 | 0.147 | -1.483 | 0.138 |
| 2‑way: Alcohol Use * Age | 0.025 | 0.023 | -0.019 | 0.069 | 1.118 | 0.264 |
| 2‑way: Sex * Age | 0.021 | 0.012 | -0.002 | 0.044 | 1.805 | 0.071 |
| 3-way: Alcohol Use * Sex * Age | -0.008 | 0.029 | -0.066 | 0.049 | -0.279 | 0.78 |
| Simple Effect: Yes – No, F @ 55 | -0.374 | 0.347 | -1.054 | 0.307 | -1.076 | 0.282 |
| Simple Effect: Yes – No, M @ 55 | -0.712 | 0.292 | -1.285 | -0.139 | -2.436 | 0.015 |
| Simple Effect: Yes – No, F @ 65 | -0.122 | 0.239 | -0.590 | 0.347 | -0.509 | 0.611 |
| Simple Effect: Yes – No, M @ 65 | -0.542 | 0.183 | -0.901 | -0.183 | -2.958 | 0.003 |
| Simple Effect: Yes – No, F @ 75 | 0.130 | 0.309 | -0.475 | 0.736 | 0.422 | 0.673 |
| Simple Effect: Yes – No, M @ 75 | -0.372 | 0.233 | -0.828 | 0.084 | -1.598 | 0.110 |
| **Social Isolation** |  |  |  |  |  |  |
| Intercept | 16.037 | 0.095 | 15.852 | 16.223 | 169.361 | <0.001 |
| Social Isolation | 0.303 | 0.217 | -0.122 | 0.728 | 1.397 | 0.163 |
| Sex | -0.88 | 0.128 | -1.131 | -0.629 | -6.875 | <0.001 |
| Age | -0.128 | 0.008 | -0.145 | -0.112 | -15.219 | <0.001 |
| Age^2^ | -0.004 | <0.001 | -0.005 | -0.003 | -7.183 | <0.001 |
| 2‑way: Isolation * Sex | 0.101 | 0.353 | -0.590 | 0.792 | 0.286 | 0.775 |
| 2‑way: Isolation * Age | 0.011 | 0.021 | -0.030 | 0.052 | 0.513 | 0.608 |
| 2‑way: Sex * Age | 0.008 | 0.014 | -0.019 | 0.035 | 0.593 | 0.553 |
| 3-way: Isolation * Sex * Age | 0.011 | 0.035 | -0.059 | 0.080 | 0.306 | 0.760 |
| Simple Effect: Yes – No, F @ 55 | 0.150 | 0.337 | -0.511 | 0.811 | 0.446 | 0.656 |
| Simple Effect: Yes – No, M @ 55 | 0.097 | 0.492 | -0.868 | 1.062 | 0.197 | 0.844 |
| Simple Effect: Yes – No, F @ 65 | 0.257 | 0.22 | -0.173 | 0.688 | 1.171 | 0.242 |
| Simple Effect: Yes – No, M @ 65 | 0.312 | 0.303 | -0.282 | 0.907 | 1.03 | 0.303 |
| Simple Effect: Yes – No, F @ 75 | 0.365 | 0.265 | -0.155 | 0.885 | 1.374 | 0.169 |
| Simple Effect: Yes – No, M @ 75 | 0.528 | 0.324 | -0.107 | 1.162 | 1.631 | 0.103 |
| **Poor Vision** |  |  |  |  |  |  |
| Intercept | 15.795 | 0.065 | 15.668 | 15.923 | 242.444 | <0.001 |
| Poor Vision | -0.725 | 0.157 | -1.033 | -0.418 | -4.625 | <0.001 |
| Sex | -0.712 | 0.092 | -0.893 | -0.531 | -7.714 | <0.001 |
| Age | -0.163 | 0.006 | -0.176 | -0.151 | -25.204 | <0.001 |
| Age^2^ | -0.004 | <0.001 | -0.005 | -0.003 | -10.576 | <0.001 |
| 2‑way: Poor Vision * Sex | 0.143 | 0.245 | -0.337 | 0.623 | 0.584 | 0.559 |
| 2‑way: Poor Vision * Age | 0.051 | 0.015 | 0.021 | 0.081 | 3.368 | 0.001 |
| 2‑way: Sex * Age | 0.021 | 0.011 | -0.002 | 0.043 | 1.812 | 0.070 |
| 3-way: Poor Vision * Sex * Age | 0.003 | 0.023 | -0.042 | 0.049 | 0.150 | 0.881 |
| Simple Effect: Yes – No, F @ 55 | -1.453 | 0.341 | -2.121 | -0.786 | -4.268 | <0.001 |
| Simple Effect: Yes – No, M @ 55 | -1.360 | 0.434 | -2.211 | -0.509 | -3.134 | 0.002 |
| Simple Effect: Yes – No, F @ 65 | -0.942 | 0.205 | -1.344 | -0.540 | -4.596 | <0.001 |
| Simple Effect: Yes – No, M @ 65 | -0.814 | 0.259 | -1.321 | -0.306 | -3.140 | 0.002 |
| Simple Effect: Yes – No, F @ 75 | -0.430 | 0.119 | -0.664 | -0.197 | -3.615 | <0.001 |
| Simple Effect: Yes – No, M @ 75 | -0.267 | 0.148 | -0.557 | 0.023 | -1.804 | 0.071 |
| **Poor Sleep** |  |  |  |  |  |  |
| Intercept | 15.86 | 0.065 | 15.732 | 15.987 | 244.181 | <0.001 |
| Poor Sleep | -0.588 | 0.088 | -0.761 | -0.414 | -6.646 | <0.001 |
| Sex | -0.690 | 0.090 | -0.867 | -0.513 | -7.632 | <0.001 |
| Age | -0.163 | 0.006 | -0.174 | -0.152 | -29.323 | <0.001 |
| Age^2^ | -0.003 | <0.001 | -0.004 | -0.003 | -13.668 | <0.001 |
| 2‑way: Poor Sleep * Sex | 0.124 | 0.141 | -0.152 | 0.400 | 0.881 | 0.378 |
| 2‑way: Poor Sleep * Age | 0.011 | 0.008 | -0.005 | 0.027 | 1.304 | 0.192 |
| 2‑way: Sex * Age | 0.026 | 0.009 | 0.009 | 0.044 | 2.915 | 0.004 |
| 3-way: Poor Sleep * Sex * Age | -0.018 | 0.014 | -0.045 | 0.009 | -1.323 | 0.186 |
| Simple Effect: Yes – No, F @ 55 | -0.740 | 0.140 | -1.015 | -0.464 | -5.267 | <0.001 |
| Simple Effect: Yes – No, M @ 55 | -0.357 | 0.188 | -0.726 | 0.012 | -1.899 | 0.058 |
| Simple Effect: Yes – No, F @ 65 | -0.633 | 0.092 | -0.814 | -0.452 | -6.854 | <0.001 |
| Simple Effect: Yes – No, M @ 65 | -0.432 | 0.117 | -0.662 | -0.202 | -3.682 | <0.001 |
| Simple Effect: Yes – No, F @ 75 | -0.526 | 0.104 | -0.729 | -0.323 | -5.083 | <0.001 |
| Simple Effect: Yes – No, M @ 75 | -0.507 | 0.129 | -0.760 | -0.255 | -3.935 | <0.001 |
| **Composite Risk Score** |  |  |  |  |  |  |
| Intercept | 17.946 | 0.097 | 17.756 | 18.136 | 185.369 | <0.001 |
| Composite Risk Score | -0.680 | 0.024 | -0.727 | -0.634 | -28.64 | <0.001 |
| Sex | -0.971 | 0.148 | -1.262 | -0.680 | -6.548 | <0.001 |
| Age | -0.146 | 0.009 | -0.163 | -0.129 | -16.552 | <0.001 |
| Age^2^ | -0.004 | <0.001 | -0.004 | -0.003 | -15.364 | <0.001 |
| 2‑way: Composite Risk Score * Sex | 0.093 | 0.039 | 0.018 | 0.169 | 2.416 | 0.016 |
| 2‑way: Composite Risk Score * Age | 0.003 | 0.002 | -0.001 | 0.008 | 1.495 | 0.135 |
| 2‑way: Sex * Age | 0.003 | 0.015 | -0.026 | 0.032 | 0.229 | 0.819 |
| 3-way: Composite Risk Score * Sex * Age | 0.003 | 0.004 | -0.005 | 0.010 | 0.649 | 0.516 |
| Simple Effect: Δ Slopes F – M @ 55 yrs | -0.058 | -0.903 | -0.183 | 0.067 | 0.367 | -0.058 |
| Simple Effect: Δ Slopes F – M @ 65 yrs | -0.083 | -2.050 | -0.162 | -0.004 | 0.04 | -0.083 |
| Simple Effect: Δ Slopes F – M @ 75 yrs | -0.108 | -2.314 | -0.199 | -0.016 | 0.021 | -0.108 |
| **Adjusted Composite Risk Score** |  |  |  |  |  |  |
| Intercept | 17.526 | 0.087 | 17.356 | 17.696 | 202.036 | <0.001 |
| Adjusted Composite Risk Score | -7.675 | 0.278 | -8.219 | -7.131 | -27.644 | <0.001 |
| Sex | -0.717 | 0.136 | -0.984 | -0.451 | -5.274 | <0.001 |
| Age | -0.146 | 0.008 | -0.161 | -0.13 | -18.538 | <0.001 |
| Age^2^ | -0.004 | <0.001 | -0.004 | -0.003 | -14.704 | <0.001 |
| 2‑way: Adjusted Composite Risk Score * Sex | 0.576 | 0.458 | -0.322 | 1.474 | 1.257 | 0.209 |
| 2‑way: Adjusted Composite Risk Score * Age | 0.053 | 0.026 | 0.002 | 0.105 | 2.017 | 0.044 |
| 2‑way: Sex * Age | 0.019 | 0.014 | -0.007 | 0.046 | 1.436 | 0.151 |
| 3-way: Adjusted Composite Risk Score * Sex * Age | -0.025 | 0.045 | -0.113 | 0.063 | -0.560 | 0.576 |
| Simple Effect: Δ Slopes F – M @ 55 yrs | -0.935 | 0.744 | -2.393 | 0.524 | -1.256 | 0.209 |
| Simple Effect: Δ Slopes F – M @ 65 yrs | -0.683 | 0.476 | -1.616 | 0.250 | -1.434 | 0.151 |
| Simple Effect: Δ Slopes F – M @ 75 yrs | -0.431 | 0.552 | -1.512 | 0.650 | -0.781 | 0.435 |

**Table 2. Reduced models for all two-way interactions between risk factor and sex on cognition for the 13 risk factors**

Interactions were further examined by sex, testing for mean differences (or linear relationships for continuous variables) in cognition between levels of the risk factor (yes vs. no) separately for women and men. Age and age^2^ were mean centered. The p-values are shown unadjusted, but we indicate with a * which simple effect results survive correction for multiple comparisons using the Bonferroni method (2 separate tests per risk factor, adjusted alpha 0.05/2=0.025). Betas are unstandardized.

| **Model Terms** | **Estimate (β)** | **Standard Error** | **95% CI**  **lower** | **95% CI**  **upper** | **t-value / t-ratio** | **p-value** |
| --- | --- | --- | --- | --- | --- | --- |
| **Education** |  |  |  |  |  |  |
| Intercept | 7.799 | 0.168 | 7.471 | 8.128 | 46.485 | <0.001 |
| Education | 0.631 | 0.013 | 0.605 | 0.656 | 48.540 | <0.001 |
| Sex | 0.236 | 0.251 | -0.256 | 0.728 | 0.942 | 0.346 |
| Age | -0.123 | 0.003 | -0.129 | -0.117 | -41.405 | <0.001 |
| Age^2^ | -0.004 | <0.001 | -0.004 | -0.003 | -15.954 | <0.001 |
| 2‑way: Education * Sex | -0.084 | 0.019 | -0.122 | -0.047 | -4.374 | <0.001* |
| Slope in Women | 0.631 | 0.013 | 0.605 | 0.656 | 48.540 | <0.001* |
| Slope in Men | 0.546 | 0.014 | 0.518 | 0.575 | 38.020 | <0.001* |
| **Poor Hearing** |  |  |  |  |  |  |
| Intercept | 16.208 | 0.068 | 16.075 | 16.34 | 239.804 | <0.001 |
| Hearing | -1.226 | 0.088 | -1.398 | -1.054 | -13.976 | <0.001 |
| Sex | -0.806 | 0.107 | -1.016 | -0.596 | -7.527 | <0.001 |
| Age | -0.140 | 0.003 | -0.147 | -0.134 | -42.779 | <0.001 |
| Age^2^ | -0.003 | <0.001 | -0.004 | -0.003 | -13.953 | <0.001 |
| 2‑way: Hearing * Sex | 0.573 | 0.140 | 0.298 | 0.848 | 4.088 | <0.001* |
| Simple Effect: Yes – No, F | -1.226 | 0.088 | -1.398 | -1.054 | -13.976 | <0.001* |
| Simple Effect: Yes – No, M | -0.652 | 0.111 | -0.870 | -0.435 | -5.885 | <0.001* |
| **Total Cholesterol** |  |  |  |  |  |  |
| Intercept | 14.588 | 0.347 | 13.908 | 15.269 | 42.028 | <0.001 |
| Cholesterol | 0.005 | 0.002 | 0.001 | 0.008 | 2.816 | 0.005 |
| Sex | 0.441 | 0.545 | -0.626 | 1.509 | 0.811 | 0.418 |
| Age | -0.145 | 0.005 | -0.156 | -0.135 | -27.294 | <0.001 |
| Age^2^ | -0.003 | <0.001 | -0.004 | -0.002 | -7.941 | <0.001 |
| 2‑way: Cholesterol * Sex | -0.005 | 0.003 | -0.011 | <-0.001 | -1.966 | 0.049* |
| Slope in Women | 0.005 | 0.002 | 0.001 | 0.008 | 2.816 | 0.005* |
| Slope in Men | -0.001 | 0.002 | -0.005 | 0.004 | -0.339 | 0.735 |
| **Depression** |  |  |  |  |  |  |
| Intercept | 15.771 | 0.056 | 15.661 | 15.881 | 281.283 | <0.001 |
| Depression | -0.917 | 0.116 | -1.144 | -0.689 | -7.896 | <0.001 |
| Sex | -0.66 | 0.074 | -0.805 | -0.516 | -8.954 | <0.001 |
| Age | -0.151 | 0.003 | -0.157 | -0.145 | -46.496 | <0.001 |
| Age^2^ | -0.004 | <0.001 | -0.004 | -0.003 | -14.145 | <0.001 |
| 2‑way: Depression * Sex | -0.236 | 0.216 | -0.660 | 0.188 | -1.090 | 0.276 |
| Simple Effect: Yes – No, F | -0.917 | 0.116 | -1.144 | -0.689 | -7.896 | <0.001 |
| Simple Effect: Yes – No, M | -1.153 | 0.183 | -1.511 | -0.794 | -6.302 | <0.001 |
| **Physical Inactivity** |  |  |  |  |  |  |
| Intercept | 14.877 | 0.07 | 14.739 | 15.015 | 211.365 | <0.001 |
| Physical Inactivity | 1.325 | 0.087 | 1.154 | 1.495 | 15.224 | <0.001 |
| Sex | -0.721 | 0.104 | -0.925 | -0.516 | -6.898 | <0.001 |
| Age | -0.141 | 0.003 | -0.148 | -0.135 | -43.731 | <0.001 |
| Age^2^ | -0.003 | <0.001 | -0.004 | -0.003 | -13.394 | <0.001 |
| 2‑way: Physical Inactivity * Sex | 0.043 | 0.138 | -0.227 | 0.314 | 0.315 | 0.753 |
| Simple Effect: Yes – No, F | -1.325 | 0.087 | -1.495 | -1.154 | -15.224 | <0.001 |
| Simple Effect: Yes – No, M | -1.368 | 0.108 | -1.580 | -1.157 | -12.691 | <0.001 |
| **Current Smoker** |  |  |  |  |  |  |
| Intercept | 15.791 | 0.080 | 15.634 | 15.949 | 196.43 | <0.001 |
| Smoker | -1.134 | 0.142 | -1.413 | -0.856 | -7.979 | <0.001 |
| Sex | -0.749 | 0.102 | -0.948 | -0.550 | -7.371 | <0.001 |
| Age | -0.157 | 0.005 | -0.166 | -0.148 | -34.202 | <0.001 |
| Age^2^ | -0.003 | <0.001 | -0.004 | -0.003 | -10.192 | <0.001 |
| 2‑way: Smoker * Sex | -0.240 | 0.212 | -0.655 | 0.176 | -1.132 | 0.258 |
| Simple Effect: Yes – No, F | -1.134 | 0.142 | -1.413 | -0.856 | -7.979 | <0.001 |
| Simple Effect: Yes – No, M | -1.374 | 0.162 | -1.693 | -1.056 | -8.460 | <0.001 |
| **Diabetes** |  |  |  |  |  |  |
| Intercept | 16.013 | 0.058 | 15.899 | 16.127 | 276.405 | <0.001 |
| Diabetes | -1.708 | 0.108 | -1.919 | -1.497 | -15.874 | <0.001 |
| Sex | -0.838 | 0.078 | -0.991 | -0.684 | -10.711 | <0.001 |
| Age | -0.147 | 0.003 | -0.153 | -0.140 | -45.285 | <0.001 |
| Age^2^ | -0.004 | <0.001 | -0.004 | -0.003 | -15.796 | <0.001 |
| 2‑way: Diabetes * Sex | 1.119 | 0.164 | 0.797 | 1.441 | 6.811 | <0.001* |
| Simple Effect: Yes – No, F | -1.708 | 0.108 | -1.919 | -1.497 | -15.874 | <0.001* |
| Simple Effect: Yes – No, M | -0.590 | 0.125 | -0.834 | -0.346 | -4.735 | <0.001* |
| **Hypertension** |  |  |  |  |  |  |
| Intercept | 16.316 | 0.079 | 16.161 | 16.471 | 206.574 | <0.001 |
| Hypertension | -1.055 | 0.091 | -1.234 | -0.875 | -11.54 | <0.001 |
| Sex | -0.814 | 0.111 | -1.031 | -0.597 | -7.347 | <0.001 |
| Age | -0.139 | 0.003 | -0.146 | -0.132 | -41.801 | <0.001 |
| Age^2^ | -0.004 | <0.001 | -0.004 | -0.003 | -15.409 | <0.001 |
| 2‑way: Hypertension * Sex | 0.294 | 0.142 | 0.017 | 0.572 | 2.077 | 0.038* |
| Simple Effect: Yes – No, F | -1.055 | 0.091 | -1.234 | -0.875 | -11.54 | <0.001* |
| Simple Effect: Yes – No, M | -0.760 | 0.110 | -0.976 | -0.545 | -6.927 | <0.001* |
| **BMI** |  |  |  |  |  |  |
| Intercept | 16.356 | 0.311 | 15.747 | 16.966 | 52.647 | <0.001 |
| BMI | -0.024 | 0.010 | -0.044 | -0.005 | -2.419 | 0.016 |
| Sex | -2.247 | 0.559 | -3.343 | -1.151 | -4.019 | <0.001 |
| Age | -0.139 | 0.005 | -0.149 | -0.129 | -27.22 | <0.001 |
| Age^2^ | -0.003 | <0.001 | -0.004 | -0.002 | -7.919 | <0.001 |
| 2‑way: BMI * Sex | 0.056 | 0.019 | 0.019 | 0.093 | 2.969 | 0.003* |
| Slope in Women | 0.024 | 0.01 | -0.044 | -0.005 | -2.419 | 0.016* |
| Slope in Men | 0.031 | 0.016 | <0.001 | 0.063 | 1.958 | 0.05 |
| **Alcohol Use** |  |  |  |  |  |  |
| Intercept | 16.900 | 0.096 | 16.711 | 17.088 | 175.786 | <0.001 |
| Alcohol Use | -0.091 | 0.237 | -0.556 | 0.374 | -0.383 | 0.702 |
| Sex | -1.031 | 0.118 | -1.262 | -0.800 | -8.745 | <0.001 |
| Age | -0.130 | 0.005 | -0.141 | -0.120 | -24.26 | <0.001 |
| Age^2^ | -0.004 | <0.001 | -0.005 | -0.003 | -9.402 | <0.001 |
| 2‑way: Alcohol Use * Sex | -0.425 | 0.297 | -1.007 | 0.157 | -1.431 | 0.153 |
| Simple Effect: Yes – No, F | -0.091 | 0.237 | -0.556 | 0.374 | -0.383 | 0.702 |
| Simple Effect: Yes – No, M | -0.516 | 0.179 | -0.866 | -0.166 | -2.888 | 0.004 |
| **Social Isolation** |  |  |  |  |  |  |
| Intercept | 16.04 | 0.095 | 15.855 | 16.226 | 169.64 | <0.001 |
| Social Isolation | 0.286 | 0.214 | -0.133 | 0.705 | 1.339 | 0.181 |
| Sex | -0.886 | 0.128 | -1.136 | -0.635 | -6.931 | <0.001 |
| Age | -0.123 | 0.006 | -0.135 | -0.111 | -19.991 | <0.001 |
| Age^2^ | -0.004 | <0.001 | -0.005 | -0.003 | -7.133 | <0.001 |
| 2‑way: Social Isolation * Sex | 0.117 | 0.351 | -0.570 | 0.805 | 0.335 | 0.738 |
| Simple Effect: Yes – No, F | 0.286 | 0.214 | -0.133 | 0.705 | 1.339 | 0.181 |
| Simple Effect: Yes – No, M | 0.403 | 0.278 | -0.142 | 0.949 | 1.449 | 0.147 |
| **Poor Vision** |  |  |  |  |  |  |
| Intercept | 15.734 | 0.064 | 15.609 | 15.859 | 247.34 | <0.001 |
| Poor Vision | -0.437 | 0.115 | -0.661 | -0.212 | -3.813 | <0.001 |
| Sex | -0.689 | 0.092 | -0.869 | -0.509 | -7.500 | <0.001 |
| Age | -0.150 | 0.005 | -0.161 | -0.140 | -28.775 | <0.001 |
| Age^2^ | -0.003 | <0.001 | -0.004 | -0.003 | -9.664 | <0.001 |
| 2‑way: Poor Vision * Sex | 0.321 | 0.173 | -0.017 | 0.660 | 1.862 | 0.063 |
| Simple Effect: Yes – No, F | -0.437 | 0.115 | -0.661 | -0.212 | -3.813 | <0.001 |
| Simple Effect: Yes – No, M | -0.115 | 0.139 | -0.388 | 0.158 | -0.828 | 0.408 |
| **Poor Sleep** |  |  |  |  |  |  |
| Intercept | 15.856 | 0.065 | 15.728 | 15.983 | 244.236 | <0.001 |
| Poor Sleep | -0.581 | 0.088 | -0.754 | -0.409 | -6.609 | <0.001 |
| Sex | -0.677 | 0.090 | -0.854 | -0.500 | -7.498 | <0.001 |
| Age | -0.152 | 0.003 | -0.158 | -0.145 | -46.436 | <0.001 |
| Age^2^ | -0.003 | <0.001 | -0.004 | -0.003 | -13.769 | <0.001 |
| 2‑way: Poor Sleep * Sex | 0.101 | 0.140 | -0.173 | 0.375 | 0.724 | 0.469 |
| Simple Effect: Yes – No, F | -0.581 | 0.088 | -0.754 | -0.409 | -6.609 | <0.001 |
| Simple Effect: Yes – No, M | -0.480 | 0.109 | -0.694 | -0.266 | -4.398 | <0.001 |
| **Composite Risk Score** |  |  |  |  |  |  |
| Intercept | 17.982 | 0.095 | 17.796 | 18.169 | 189.246 | <0.001 |
| Composite Risk Score | -0.691 | 0.023 | -0.736 | -0.644 | -29.554 | <0.001 |
| Sex | -1.010 | 0.144 | -1.292 | -0.727 | -6.997 | <0.001 |
| Age | -0.130 | 0.003 | -0.136 | -0.124 | -40.872 | <0.001 |
| Age^2^ | -0.004 | <0.001 | -0.004 | -0.003 | -15.169 | <0.001 |
| 2‑way: Composite Risk Score * Sex | 0.105 | 0.038 | 0.031 | 0.179 | 2.776 | 0.005* |
| Slope in Women | -0.690 | 0.023 | -0.736 | -0.644 | -29.554 | <0.001* |
| Slope in Men | -0.585 | 0.030 | -0.644 | -0.526 | -19.437 | <0.001* |
| **Adjusted Composite Risk Score** |  |  |  |  |  |  |
| Intercept | 17.567 | 0.085 | 17.399 | 17.734 | 205.876 | <0.001 |
| Adjusted Composite Risk Score | -7.814 | 0.273 | -8.349 | -7.278 | -28.595 | <0.001 |
| Sex | -0.783 | 0.131 | -1.041 | -0.526 | -5.964 | <0.001 |
| Age | -0.127 | 0.003 | -0.134 | -0.121 | -39.795 | <0.001 |
| Age^2^ | -0.004 | <0.001 | -0.004 | -0.003 | -14.546 | <0.001 |
| 2‑way: Adjusted Composite Risk Score * Sex | 0.788 | 0.447 | -0.088 | 1.664 | 1.764 | 0.078 |
| Slope in Women | -7.814 | 0.273 | -8.349 | -7.278 | -28.595 | <0.001 |
| Slope in Men | -7.025 | 0.359 | -7.728 | -6.322 | -19.583 | <0.001 |

**Table 3. Three-way interactions between risk factor, age group, and sex on cognition, with education as a covariate, for the 13 risk factors.** Each three-way interaction was probed at three representative ages (55, 65, and 75), testing two-way interactions between risk factor and sex on cognition. Age, age^2,^ and education were mean centered. The model testing the two-way interaction of education and sex on cognition was excluded, since education was added as a covariate to these models. The p-values are shown unadjusted. Betas are unstandardized.

| **Model Terms** | **Estimate (β)** | **Standard Error** | **95% CI**  **lower** | **95% CI**  **upper** | **t-value / t-ratio** | **p-value** |
| --- | --- | --- | --- | --- | --- | --- |
| **Poor Hearing** |  |  |  |  |  |  |
| Intercept | 15.973 | 0.062 | 15.852 | 16.094 | 258.045 | <0.001 |
| Hearing | -0.675 | 0.081 | -0.834 | -0.517 | -8.362 | <0.001 |
| Sex | -1.047 | 0.100 | -1.243 | -0.851 | -10.457 | <0.001 |
| Age | -0.136 | 0.006 | -0.147 | -0.126 | -24.668 | <0.001 |
| Age^2^ | -0.004 | <0.001 | -0.004 | -0.003 | -16.085 | <0.001 |
| Education | 0.581 | 0.010 | 0.562 | 0.600 | 59.47 | <0.001 |
| 2‑way: Hearing * Sex | 0.515 | 0.130 | 0.260 | 0.770 | 3.963 | <0.001 |
| 2‑way: Hearing * Age | 0.022 | 0.008 | 0.007 | 0.037 | 2.830 | 0.005 |
| 2‑way: Sex * Age | 0.014 | 0.010 | -0.005 | 0.034 | 1.423 | 0.155 |
| 3-way: Hearing * Sex * Age | -0.003 | 0.013 | -0.028 | 0.023 | -0.203 | 0.839 |
| Simple Effect: Poor – Good, F @ 55 | -0.972 | 0.128 | -1.239 | -0.726 | -7.605 | <0.001 |
| Simple Effect: Poor – Good, M @ 55 | -0.421 | 0.169 | -0.770 | -0.091 | -2.489 | 0.013 |
| Simple Effect: Poor – Good, F @ 65 | -0.756 | 0.084 | -0.933 | -0.600 | -9.024 | <0.001 |
| Simple Effect: Poor – Good, M @ 65 | -0.231 | 0.106 | -0.452 | -0.030 | -2.180 | 0.029 |
| Simple Effect: Poor – Good, F @ 75 | -0.540 | 0.097 | -0.736 | -0.366 | -5.592 | <0.001 |
| Simple Effect: Poor – Good, M @ 75 | -0.042 | 0.128 | -0.295 | 0.193 | -0.326 | 0.745 |
| **Total Cholesterol** |  |  |  |  |  |  |
| Intercept | 15.255 | 0.314 | 14.64 | 15.871 | 48.588 | <0.001 |
| Cholesterol | 0.002 | 0.001 | -0.001 | 0.004 | 1.059 | 0.29 |
| Sex | -0.336 | 0.498 | -1.312 | 0.641 | -0.674 | 0.500 |
| Age | -0.104 | 0.030 | -0.162 | -0.045 | -3.474 | 0.001 |
| Age^2^ | -0.003 | <0.001 | -0.004 | -0.002 | -8.390 | <0.001 |
| Education | 0.591 | 0.015 | 0.561 | 0.621 | 38.444 | <0.001 |
| 2‑way: Cholesterol * Sex | -0.003 | 0.002 | -0.007 | 0.002 | -1.052 | 0.293 |
| 2‑way: Cholesterol * Age | <-0.001 | <0.001 | <-0.001 | <0.001 | -0.693 | 0.488 |
| 2‑way: Sex * Age | -0.033 | 0.050 | -0.131 | 0.065 | -0.663 | 0.508 |
| 3-way: Cholesterol * Sex * Age | <0.001 | <0.001 | <-0.001 | 0.001 | 0.641 | 0.522 |
| Simple Effect: Δ Slopes F – M @ 55 yrs | 0.005 | 0.004 | -0.003 | 0.013 | 1.169 | 0.243 |
| Simple Effect: Δ Slopes F – M @ 65 yrs | 0.003 | 0.003 | -0.002 | 0.008 | 1.253 | 0.210 |
| Simple Effect: Δ Slopes F – M @ 75 yrs | 0.002 | 0.003 | -0.004 | 0.007 | 0.570 | 0.568 |
| **Depression** |  |  |  |  |  |  |
| Intercept | 15.732 | 0.051 | 15.633 | 15.832 | 309.900 | <0.001 |
| Depression | -0.47 | 0.107 | -0.680 | -0.261 | -4.397 | <0.001 |
| Sex | -0.841 | 0.067 | -0.972 | -0.710 | -12.578 | <0.001 |
| Age | -0.133 | 0.004 | -0.141 | -0.125 | -32.982 | <0.001 |
| Age^2^ | -0.004 | <0.001 | -0.004 | -0.003 | -15.796 | <0.001 |
| Education | 0.587 | 0.010 | 0.568 | 0.606 | 60.468 | <0.001 |
| 2‑way: Depression * Sex | -0.148 | 0.199 | -0.538 | 0.243 | -0.740 | 0.459 |
| 2‑way: Depression * Age | 0.011 | 0.010 | -0.009 | 0.030 | 1.075 | 0.282 |
| 2‑way: Sex * Age | 0.017 | 0.007 | 0.004 | 0.030 | 2.546 | 0.011 |
| 3-way: Depression * Sex * Age | 0.024 | 0.019 | -0.013 | 0.061 | 1.289 | 0.197 |
| Simple Effect: Yes – No, F @ 55 | -0.617 | 0.158 | -0.940 | -0.305 | -3.894 | <0.001 |
| Simple Effect: Yes – No, M @ 55 | -1.096 | 0.254 | -1.622 | -0.603 | -4.314 | <0.001 |
| Simple Effect: Yes – No, F @ 65 | -0.510 | 0.107 | -0.727 | -0.304 | -4.764 | <0.001 |
| Simple Effect: Yes – No, M @ 65 | -0.748 | 0.170 | -1.101 | -0.429 | -4.404 | <0.001 |
| Simple Effect: Yes – No, F @ 75 | -0.403 | 0.133 | -0.663 | -0.155 | -3.044 | 0.002 |
| Simple Effect: Yes – No, M @ 75 | -0.400 | 0.208 | -0.817 | -0.018 | -1.922 | 0.055 |
| **Physical Inactivity** |  |  |  |  |  |  |
| Intercept | 15.199 | 0.064 | 15.073 | 15.325 | 236.321 | <0.001 |
| Physical Inactivity | 0.806 | 0.080 | 0.649 | 0.963 | 10.079 | <0.001 |
| Sex | -0.886 | 0.096 | -1.073 | -0.698 | -9.270 | <0.001 |
| Age | -0.132 | 0.005 | -0.142 | -0.122 | -25.197 | <0.001 |
| Age^2^ | -0.003 | <0.001 | -0.004 | -0.003 | -14.698 | <0.001 |
| Education | 0.575 | 0.010 | 0.556 | 0.594 | 59.027 | <0.001 |
| 2‑way: Physical Inactivity * Sex | 0.023 | 0.126 | -0.225 | 0.271 | 0.183 | 0.854 |
| 2‑way: Physical Inactivity * Age | 0.012 | 0.007 | -0.003 | 0.026 | 1.548 | 0.122 |
| 2‑way: Sex * Age | 0.021 | 0.009 | 0.004 | 0.039 | 2.352 | 0.019 |
| 3-way: Physical Inactivity * Sex * Age | -0.007 | 0.012 | -0.031 | 0.017 | -0.553 | 0.500 |
| Simple Effect: Yes – No, F @ 55 | -0.647 | 0.127 | -0.895 | -0.387 | 5.102 | <0.001 |
| Simple Effect: Yes – No, M @ 55 | -0.764 | 0.171 | -1.104 | -0.419 | -4.476 | <0.001 |
| Simple Effect: Yes – No, F @ 65 | -0.763 | 0.083 | -0.923 | -0.592 | -9.162 | <0.001 |
| Simple Effect: Yes – No, M @ 65 | -0.812 | 0.107 | -1.022 | -0.597 | -7.618 | <0.001 |
| Simple Effect: Yes – No, F @ 75 | -0.879 | 0.095 | -1.055 | -0.691 | -9.255 | <0.001 |
| Simple Effect: Yes – No, M @ 75 | -0.859 | 0.116 | -1.079 | -0.635 | -7.435 | <0.001 |
| **Current Smoker** |  |  |  |  |  |  |
| Intercept | 15.639 | 0.074 | 15.493 | 15.785 | 210.178 | <0.001 |
| Smoker | -0.472 | 0.144 | -0.754 | -0.190 | -3.277 | 0.001 |
| Sex | -0.759 | 0.093 | -0.942 | -0.576 | -8.146 | <0.001 |
| Age | -0.153 | 0.007 | -0.166 | -0.141 | -23.495 | <0.001 |
| Age^2^ | -0.003 | <0.001 | -0.004 | -0.002 | -9.009 | <0.001 |
| Education | 0.580 | 0.013 | 0.554 | 0.607 | 43.669 | <0.001 |
| 2‑way: Smoker * Sex | -0.112 | 0.219 | -0.542 | 0.318 | -0.509 | 0.611 |
| 2‑way: Smoker * Age | 0.053 | 0.014 | 0.026 | 0.080 | 3.810 | <0.001 |
| 2‑way: Sex * Age | 0.0270 | 0.009 | 0.008 | 0.045 | 2.853 | 0.004 |
| 3-way: Smoker * Sex * Age | -0.017 | 0.021 | -0.059 | 0.025 | -0.799 | 0.424 |
| Simple Effect: Yes – No, F @ 55 | -1.199 | 0.188 | -1.603 | -0.848 | -6.393 | <0.001 |
| Simple Effect: Yes – No, M @ 55 | -1.075 | 0.228 | -1.552 | -0.634 | -4.722 | <0.001 |
| Simple Effect: Yes – No, F @ 65 | -0.670 | 0.132 | -0.955 | -0.437 | -5.070 | <0.001 |
| Simple Effect: Yes – No, M @ 65 | -0.717 | 0.152 | -1.034 | -0.436 | -4.706 | <0.001 |
| Simple Effect: Yes – No, F @ 75 | -0.141 | 0.196 | -0.541 | 0.207 | -0.718 | 0.473 |
| Simple Effect: Yes – No, M @ 75 | -0.360 | 0.234 | -0.823 | 0.068 | -1.541 | 0.123 |
| **Diabetes** |  |  |  |  |  |  |
| Intercept | 15.899 | 0.053 | 15.796 | 16.002 | 302.369 | <0.001 |
| Diabetes | -1.073 | 0.098 | -1.265 | -0.881 | -10.947 | <0.001 |
| Sex | -0.981 | 0.071 | -1.12 | -0.842 | -13.793 | <0.001 |
| Age | -0.130 | 0.004 | -0.138 | -0.122 | -32.419 | <0.001 |
| Age^2^ | -0.004 | <0.001 | -0.004 | -0.003 | -16.969 | <0.001 |
| Education | 0.581 | 0.010 | 0.562 | 0.600 | 59.603 | <0.001 |
| 2‑way: Diabetes * Sex | 0.782 | 0.149 | 0.489 | 1.074 | 5.234 | <0.001 |
| 2‑way: Diabetes * Age | 0.005 | 0.010 | -0.015 | 0.024 | 0.455 | 0.649 |
| 2‑way: Sex * Age | 0.019 | 0.007 | 0.006 | 0.032 | 2.795 | 0.005 |
| 3-way: Diabetes * Sex * Age | 0.003 | 0.016 | -0.028 | 0.034 | 0.204 | 0.839 |
| Simple Effect: Yes – No, F @ 55 | -1.135 | 0.168 | -1.474 | -0.801 | -6.770 | <0.001 |
| Simple Effect: Yes – No, M @ 55 | -0.398 | 0.209 | -0.822 | 0.018 | -1.903 | 0.057 |
| Simple Effect: Yes – No, F @ 65 | -1.090 | 0.105 | -1.301 | -0.883 | -10.412 | <0.001 |
| Simple Effect: Yes – No, M @ 65 | -0.320 | 0.125 | -0.574 | -0.074 | -2.564 | 0.010 |
| Simple Effect: Yes – No, F @ 75 | -1.045 | 0.116 | -1.270 | -0.824 | -8.984 | <0.001 |
| Simple Effect: Yes – No, M @ 75 | -0.243 | 0.133 | -0.502 | 0.008 | -1.824 | 0.068 |
| **Hypertension** |  |  |  |  |  |  |
| Intercept | 16.060 | 0.073 | 15.917 | 16.203 | 220.526 | <0.001 |
| Hypertension | -0.613 | 0.085 | -0.779 | -0.447 | -7.254 | <0.001 |
| Sex | -0.891 | 0.103 | -1.093 | -0.688 | -8.633 | <0.001 |
| Age | -0.134 | 0.006 | -0.146 | -0.122 | -22.556 | <0.001 |
| Age^2^ | -0.004 | <0.001 | -0.004 | -0.004 | -17.033 | <0.001 |
| Education | 0.585 | 0.010 | 0.566 | 0.604 | 60.014 | <0.001 |
| 2‑way: Hypertension * Sex | 0.079 | 0.131 | -0.178 | 0.335 | 0.601 | 0.548 |
| 2‑way: Hypertension * Age | 0.018 | 0.008 | 0.003 | 0.034 | 2.334 | 0.020 |
| 2‑way: Sex * Age | 0.005 | 0.010 | -0.014 | 0.024 | 0.492 | 0.623 |
| 3-way: Hypertension * Sex * Age | 0.022 | 0.013 | -0.003 | 0.047 | 1.731 | 0.084 |
| Simple Effect: Yes – No, F @ 55 | -0.864 | 0.127 | -1.128 | -0.618 | -6.791 | <0.001 |
| Simple Effect: Yes – No, M @ 55 | -1.088 | 0.168 | -1.444 | -0.771 | -6.489 | <0.001 |
| Simple Effect: Yes – No, F @ 65 | -0.681 | 0.086 | -0.86 | -0.521 | -7.964 | <0.001 |
| Simple Effect: Yes – No, M @ 65 | -0.685 | 0.105 | -0.915 | -0.495 | -6.497 | <0.001 |
| Simple Effect: Yes – No, F @ 75 | -0.499 | 0.103 | -0.706 | -0.310 | -4.828 | <0.001 |
| Simple Effect: Yes – No, M @ 75 | -0.282 | 0.121 | -0.534 | -0.070 | -2.329 | 0.020 |
| **BMI** |  |  |  |  |  |  |
| Intercept | 15.616 | 0.283 | 15.061 | 16.172 | 55.118 | <0.001 |
| BMI | 0.004 | 0.009 | -0.014 | 0.022 | 0.452 | 0.651 |
| Sex | -2.003 | 0.509 | -3.001 | -1.005 | -3.935 | <0.001 |
| Age | -0.206 | 0.026 | -0.256 | -0.155 | -8.023 | <0.001 |
| Age^2^ | -0.003 | <0.001 | -0.004 | -0.002 | -7.694 | <0.001 |
| Education | 0.588 | 0.015 | 0.560 | 0.617 | 39.978 | <0.001 |
| 2‑way: BMI * Sex | 0.037 | 0.017 | 0.004 | 0.071 | 2.182 | 0.029 |
| 2‑way: BMI * Age | 0.003 | 0.001 | 0.001 | 0.005 | 3.475 | 0.001 |
| 2‑way: Sex * Age | 0.177 | 0.050 | 0.079 | 0.276 | 3.525 | <0.001 |
| 3-way: BMI * Sex * Age | -0.006 | 0.002 | -0.009 | -0.002 | -3.387 | 0.001* |
| Simple Effect: Δ Slopes F – M @ 55 yrs | -0.118 | 0.029 | -0.174 | -0.062 | -4.099 | <0.001* |
| Simple Effect: Δ Slopes F – M @ 65 yrs | -0.059 | 0.018 | -0.095 | -0.024 | -3.281 | 0.001* |
| Simple Effect: Δ Slopes F – M @ 75 yrs | -0.001 | 0.021 | -0.041 | 0.040 | -0.030 | 0.976 |
| **Alcohol Use** |  |  |  |  |  |  |
| Intercept | 16.248 | 0.092 | 16.067 | 16.429 | 175.891 | <0.001 |
| Alcohol Use | -0.077 | 0.228 | -0.524 | 0.371 | -0.335 | 0.737 |
| Sex | -1.027 | 0.111 | -1.245 | -0.809 | -9.223 | <0.001 |
| Age | -0.130 | 0.007 | -0.145 | -0.116 | -17.605 | <0.001 |
| Age^2^ | -0.004 | <0.001 | -0.004 | -0.003 | -9.172 | <0.001 |
| Education | 0.545 | 0.018 | 0.510 | 0.580 | 30.584 | <0.001 |
| 2‑way: Alcohol Use * Sex | -0.125 | 0.284 | -0.683 | 0.432 | -0.441 | 0.659 |
| 2‑way: Alcohol Use * Age | 0.018 | 0.021 | -0.023 | 0.059 | 0.862 | 0.389 |
| 2‑way: Sex * Age | 0.025 | 0.011 | 0.004 | 0.047 | 2.344 | 0.019 |
| 3-way: Alcohol Use * Sex * Age | -0.019 | 0.027 | -0.073 | 0.034 | -0.707 | 0.480 |
| Simple Effect: Yes – No, F @ 55 | -0.326 | 0.319 | -0.974 | 0.304 | -1.024 | 0.306 |
| Simple Effect: Yes – No, M @ 55 | -0.184 | 0.267 | -0.720 | 0.352 | -0.692 | 0.489 |
| Simple Effect: Yes – No, F @ 65 | -0.144 | 0.221 | -0.589 | 0.283 | -0.653 | 0.514 |
| Simple Effect: Yes – No, M @ 65 | -0.197 | 0.169 | -0.531 | 0.138 | -1.167 | 0.243 |
| Simple Effect: Yes – No, F @ 75 | 0.037 | 0.292 | -0.531 | 0.587 | 0.127 | 0.899 |
| Simple Effect: Yes – No, M @ 75 | -0.210 | 0.222 | -0.633 | 0.214 | -0.947 | 0.344 |
| **Social Isolation** |  |  |  |  |  |  |
| Intercept | 15.944 | 0.086 | 15.776 | 16.113 | 185.522 | <0.001 |
| Social Isolation | 0.290 | 0.196 | -0.094 | 0.674 | 1.479 | 0.139 |
| Sex | -1.126 | 0.117 | -1.354 | -0.897 | -9.661 | <0.001 |
| Age | -0.115 | 0.008 | -0.130 | -0.100 | -14.987 | <0.001 |
| Age^2^ | -0.004 | <0.001 | -0.004 | -0.003 | -7.793 | <0.001 |
| Education | 0.587 | 0.018 | 0.551 | 0.622 | 32.208 | <0.001 |
| 2‑way: Isolation * Sex | 0.222 | 0.319 | -0.404 | 0.848 | 0.694 | 0.487 |
| 2‑way: Isolation * Age | 0.029 | 0.019 | -0.008 | 0.066 | 1.545 | 0.122 |
| 2‑way: Sex * Age | 0.019 | 0.013 | -0.006 | 0.044 | 1.518 | 0.129 |
| 3-way: Isolation * Sex * Age | -0.018 | 0.032 | -0.725 | 0.472 | -0.545 | 0.586 |
| Simple Effect: Yes – No, F @ 55 | -0.112 | 0.298 | -0.536 | 1.227 | -0.375 | 0.708 |
| Simple Effect: Yes – No, M @ 55 | 0.351 | 0.439 | -0.224 | 0.556 | 0.800 | 0.424 |
| Simple Effect: Yes – No, F @ 65 | 0.180 | 0.197 | -0.079 | 1.003 | 0.916 | 0.360 |
| Simple Effect: Yes – No, M @ 65 | 0.468 | 0.271 | -0.011 | 0.928 | 1.726 | 0.084 |
| Simple Effect: Yes – No, F @ 75 | 0.473 | 0.245 | 0.004 | 1.154 | 1.928 | 0.054 |
| Simple Effect: Yes – No, M @ 75 | 0.585 | 0.300 | -0.725 | 0.472 | 1.948 | 0.052 |
| **Poor Vision** |  |  |  |  |  |  |
| Intercept | 15.802 | 0.059 | 15.687 | 15.917 | 269.392 | <0.001 |
| Poor Vision | -0.444 | 0.141 | -0.721 | -0.168 | -3.146 | 0.002 |
| Sex | -0.900 | 0.083 | -1.063 | -0.737 | -10.812 | <0.001 |
| Age | -0.144 | 0.006 | -0.155 | -0.132 | -24.601 | <0.001 |
| Age^2^ | -0.004 | <0.001 | -0.005 | -0.003 | -11.187 | <0.001 |
| Education | 0.592 | 0.011 | 0.571 | 0.613 | 55.443 | <0.001 |
| 2‑way: Poor Vision * Sex | -0.023 | 0.221 | -0.456 | 0.410 | -0.102 | 0.918 |
| 2‑way: Poor Vision * Age | 0.036 | 0.014 | 0.009 | 0.062 | 2.604 | 0.009 |
| 2‑way: Sex * Age | 0.027 | 0.010 | 0.007 | 0.047 | 2.636 | 0.008 |
| 3-way: Poor Vision * Sex * Age | 0.007 | 0.021 | -0.034 | 0.048 | 0.322 | 0.747 |
| Simple Effect: Yes – No, F @ 55 | -0.933 | 0.300 | -1.552 | -0.350 | -3.108 | 0.002 |
| Simple Effect: Yes – No, M @ 55 | -1.049 | 0.383 | -1.838 | -0.302 | -2.736 | 0.006 |
| Simple Effect: Yes – No, F @ 65 | -0.578 | 0.179 | -0.957 | -0.233 | -3.224 | 0.001 |
| Simple Effect: Yes – No, M @ 65 | -0.625 | 0.227 | -1.105 | -0.188 | -2.759 | 0.006 |
| Simple Effect: Yes – No, F @ 75 | -0.222 | 0.107 | -0.449 | -0.029 | -2.080 | 0.038 |
| Simple Effect: Yes – No, M @ 75 | -0.202 | 0.133 | -0.485 | 0.039 | -1.520 | 0.129 |
| **Poor Sleep** |  |  |  |  |  |  |
| Intercept | 15.755 | 0.059 | 15.64 | 15.871 | 268.087 | <0.001 |
| Poor Sleep | -0.255 | 0.080 | -0.412 | -0.098 | -3.182 | 0.001 |
| Sex | -0.884 | 0.082 | -1.045 | -0.724 | -10.790 | <0.001 |
| Age | -0.137 | 0.005 | -0.147 | -0.127 | -26.921 | <0.001 |
| Age^2^ | -0.004 | <0.001 | -0.004 | -0.003 | -15.412 | <0.001 |
| Education | 0.590 | 0.010 | 0.571 | 0.609 | 60.672 | <0.001 |
| 2‑way: Poor Sleep * Sex | 0.115 | 0.127 | -0.135 | 0.364 | 0.900 | 0.368 |
| 2‑way: Poor Sleep * Age | 0.011 | 0.007 | -0.003 | 0.026 | 1.493 | 0.135 |
| 2‑way: Sex * Age | 0.026 | 0.008 | 0.010 | 0.042 | 3.147 | 0.002 |
| 3-way: Poor Sleep * Sex * Age | -0.012 | 0.012 | -0.036 | 0.013 | -0.953 | 0.340 |
| Simple Effect: Yes – No, F @ 55 | -0.407 | 0.125 | -0.664 | -0.162 | -3.255 | 0.001 |
| Simple Effect: Yes – No, M @ 55 | -0.129 | 0.167 | -0.465 | 0.207 | -0.772 | 0.440 |
| Simple Effect: Yes – No, F @ 65 | -0.296 | 0.083 | -0.466 | -0.137 | -3.575 | <0.001 |
| Simple Effect: Yes – No, M @ 65 | -0.137 | 0.105 | -0.346 | 0.072 | -1.307 | 0.191 |
| Simple Effect: Yes – No, F @ 75 | -0.186 | 0.095 | -0.374 | -0.008 | -1.943 | 0.052 |
| Simple Effect: Yes – No, M @ 75 | -0.145 | 0.119 | -0.374 | 0.084 | -1.218 | 0.223 |
| **Composite Risk Score** |  |  |  |  |  |  |
| Intercept | 16.84 | 0.092 | 16.659 | 17.021 | 182.197 | <0.001 |
| Composite Risk Score | -0.349 | 0.023 | -0.394 | -0.304 | -15.196 | <0.001 |
| Sex | -1.27 | 0.138 | -1.540 | -1.00 | -9.214 | <0.001 |
| Age | -0.139 | 0.008 | -0.155 | -0.123 | -16.937 | <0.001 |
| Age^2^ | -0.004 | 0 | -0.004 | -0.003 | -16.651 | <0.001 |
| Education | 0.533 | 0.01 | 0.513 | 0.553 | 51.775 | <0.001 |
| 2‑way: Composite Risk Score * Sex | 0.131 | 0.036 | 0.061 | 0.201 | 3.65 | <0.001 |
| 2‑way: Composite Risk Score * Age | 0.005 | 0.002 | 0.001 | 0.009 | 2.347 | 0.019 |
| 2‑way: Sex * Age | 0.004 | 0.014 | -0.023 | 0.031 | 0.295 | 0.768 |
| 3-way: Composite Risk Score * Sex * Age | 0.003 | 0.004 | -0.004 | 0.010 | 0.860 | 0.390 |
| Simple Effect: Δ Slopes F – M @ 55 yrs | -0.087 | 0.060 | -0.204 | 0.030 | -1.455 | 0.146 |
| Simple Effect: Δ Slopes F – M @ 65 yrs | -0.118 | 0.038 | -0.191 | -0.044 | -3.135 | 0.002 |
| Simple Effect: Δ Slopes F – M @ 75 yrs | -0.149 | 0.043 | -0.233 | -0.064 | -3.445 | 0.001 |
| **Adjusted Composite Risk Score** |  |  |  |  |  |  |
| Intercept | 16.524 | 0.083 | 16.362 | 16.687 | 199.222 | <0.001 |
| Adjusted Composite Risk Score | -3.519 | 0.270 | -4.049 | -2.989 | -13.022 | <0.001 |
| Sex | -1.232 | 0.127 | -1.481 | -0.984 | -9.718 | <0.001 |
| Age | -0.138 | 0.007 | -0.152 | -0.123 | -18.83 | <0.001 |
| Age^2^ | -0.004 | <0.001 | -0.004 | -0.003 | -16.371 | <0.001 |
| Education | 0.540 | 0.011 | 0.519 | 0.560 | 51.366 | <0.001 |
| 2‑way: Adjusted Composite Risk Score * Sex | 1.706 | 0.426 | 0.870 | 2.542 | 4.000 | <0.001 |
| 2‑way: Adjusted Composite Risk Score * Age | 0.062 | 0.024 | 0.014 | 0.110 | 2.532 | 0.011 |
| 2‑way: Sex * Age | 0.005 | 0.013 | -0.020 | 0.030 | 0.399 | 0.690 |
| 3-way: Adjusted Composite Risk Score * Sex * Age | 0.032 | 0.042 | -0.050 | 0.115 | 0.770 | 0.441 |
| Simple Effect: Δ Slopes F – M @ 55 yrs | -1.246 | 0.696 | -2.609 | 0.118 | -1.791 | 0.073 |
| Simple Effect: Δ Slopes F – M @ 65 yrs | -1.569 | 0.444 | -2.439 | -0.699 | -3.534 | <0.001 |
| Simple Effect: Δ Slopes F – M @ 75 yrs | -1.892 | 0.513 | -2.897 | -0.887 | -3.692 | <0.001 |

**Table 4. Two-way interactions between risk factor sex on cognition, with education as a covariate, for 12 risk factors.** Interactions were further examined by sex, testing for mean differences (or linear relationships for continuous variables) in cognition between levels of the risk factor (yes vs. no) separately for women and men. The model testing the two-way interaction of education and sex on cognition was excluded, since education was added as a covariate to these models. Age, age^2^ , and education were mean centered. The p-values are shown unadjusted. Betas are unstandardized.

| **Model Terms** | **Estimate (β)** | **Standard Error** | **95% CI**  **lower** | **95% CI**  **upper** | **t-value / t-ratio** | **p-value** |
| --- | --- | --- | --- | --- | --- | --- |
| **Poor Hearing** |  |  |  |  |  |  |
| Intercept | 15.994 | 0.061 | 15.873 | 16.114 | 260.330 | <0.001 |
| Hearing | -0.714 | 0.080 | -0.871 | -0.557 | -8.910 | <0.001 |
| Sex | -1.083 | 0.097 | -1.274 | -0.892 | -11.113 | <0.001 |
| Age | -0.120 | 0.003 | -0.125 | -0.114 | -39.716 | <0.001 |
| Age^2^ | -0.004 | <0.001 | -0.004 | -0.003 | -15.668 | <0.001 |
| Education | 0.582 | 0.010 | 0.563 | 0.602 | 59.683 | <0.001 |
| 2‑way: Hearing * Sex | 0.569 | 0.127 | 0.319 | 0.819 | 4.461 | <0.001 |
| Simple Effect: Yes – No, F | -0.714 | 0.08 | -0.871 | -0.557 | -8.910 | <0.001 |
| Simple Effect: Yes – No, M | -0.145 | 0.101 | -0.343 | 0.053 | -1.432 | 0.152 |
| **Total Cholesterol** |  |  |  |  |  |  |
| Intercept | 15.242 | 0.312 | 14.63 | 15.855 | 48.791 | <0.001 |
| Cholesterol | 0.002 | 0.001 | -0.001 | 0.005 | 1.124 | 0.261 |
| Sex | -0.327 | 0.491 | -1.289 | 0.635 | -0.666 | 0.505 |
| Age | -0.124 | 0.005 | -0.134 | -0.115 | -25.676 | <0.001 |
| Age^2^ | -0.003 | <0.001 | -0.004 | -0.002 | -8.526 | <0.001 |
| Education | 0.591 | 0.015 | 0.561 | 0.621 | 38.450 | <0.001 |
| 2‑way: Cholesterol * Sex | -0.003 | 0.002 | -0.007 | 0.002 | -1.107 | 0.269 |
| Slope in Women | 0.002 | 0.001 | -0.001 | 0.005 | 1.124 | 0.261 |
| Slope in Men | -0.001 | 0.002 | -0.005 | 0.003 | -0.537 | 0.592 |
| **Depression** |  |  |  |  |  |  |
| Intercept | 15.736 | 0.051 | 15.636 | 15.835 | 310.128 | <0.001 |
| Depression | -0.478 | 0.105 | -0.684 | -0.271 | -4.532 | <0.001 |
| Sex | -0.842 | 0.067 | -0.973 | -0.711 | -12.593 | <0.001 |
| Age | -0.125 | 0.003 | -0.131 | -0.119 | -41.839 | <0.001 |
| Age^2^ | -0.004 | <0.001 | -0.004 | -0.003 | -15.861 | <0.001 |
| Education | 0.587 | 0.010 | 0.568 | 0.606 | 60.449 | <0.001 |
| 2‑way: Depression * Sex | -0.222 | 0.197 | -0.607 | 0.164 | -1.128 | 0.259 |
| Simple Effect: Yes – No, F | -0.478 | 0.105 | -0.684 | -0.271 | -4.532 | <0.001 |
| Simple Effect: Yes – No, M | -0.699 | 0.166 | -1.026 | -0.373 | -4.203 | <0.001 |
| **Physical Inactivity** |  |  |  |  |  |  |
| Intercept | 15.193 | 0.064 | 15.067 | 15.319 | 236.705 | <0.001 |
| Physical Inactivity | 0.819 | 0.080 | 0.663 | 0.975 | 10.293 | <0.001 |
| Sex | -0.864 | 0.095 | -1.050 | -0.678 | -9.091 | <0.001 |
| Age | -0.120 | 0.003 | -0.126 | -0.114 | -40.195 | <0.001 |
| Age^2^ | -0.003 | <0.001 | -0.004 | -0.003 | -15.279 | <0.001 |
| Education | 0.575 | 0.010 | 0.555 | 0.594 | 58.985 | <0.001 |
| 2‑way: Physical Inactivity * Sex | -0.011 | 0.126 | -0.257 | 0.235 | -0.089 | 0.929 |
| Simple Effect: Yes – No, F | -0.819 | 0.080 | -0.975 | -0.663 | -10.293 | <0.001 |
| Simple Effect: Yes – No, M | -0.808 | 0.099 | -1.002 | -0.615 | -8.193 | <0.001 |
| **Current Smoker** |  |  |  |  |  |  |
| Intercept | 15.675 | 0.073 | 15.532 | 15.819 | 213.573 | <0.001 |
| Smoker | -0.636 | 0.130 | -0.892 | -0.38 | -4.874 | <0.001 |
| Sex | -0.751 | 0.093 | -0.933 | -0.570 | -8.102 | <0.001 |
| Age | -0.131 | 0.004 | -0.140 | -0.123 | -31.017 | <0.001 |
| Age^2^ | -0.003 | <0.001 | -0.004 | -0.003 | -10.912 | <0.001 |
| Education | 0.581 | 0.013 | 0.554 | 0.607 | 43.631 | <0.001 |
| 2‑way: Smoker * Sex | -0.165 | 0.194 | -0.545 | 0.215 | -0.849 | 0.396 |
| Simple Effect: Yes – No, F | -0.636 | 0.130 | -0.892 | -0.380 | -4.874 | <0.001 |
| Simple Effect: Yes – No, M | -0.801 | 0.149 | -1.093 | -0.508 | -5.367 | <0.001 |
| **Diabetes** |  |  |  |  |  |  |
| Intercept | 15.902 | 0.053 | 15.799 | 16.005 | 302.452 | <0.001 |
| Diabetes | -1.079 | 0.098 | -1.271 | -0.887 | -11.004 | <0.001 |
| Sex | -0.99 | 0.071 | -1.129 | -0.850 | -13.923 | <0.001 |
| Age | -0.123 | 0.003 | -0.128 | -0.117 | -41.189 | <0.001 |
| Age^2^ | -0.004 | <0.001 | -0.004 | -0.003 | -16.868 | <0.001 |
| Education | 0.580 | 0.010 | 0.561 | 0.600 | 59.578 | <0.001 |
| 2‑way: Diabetes * Sex | 0.805 | 0.149 | 0.805 | 0.149 | 5.401 | <0.001 |
| Simple Effect: Yes – No, F | -1.079 | 0.098 | -1.271 | -0.887 | -11.004 | <0.001 |
| Simple Effect: Yes – No, M | -0.274 | 0.113 | -0.495 | -0.052 | -2.416 | 0.016 |
| **Hypertension** |  |  |  |  |  |  |
| Intercept | 16.098 | 0.072 | 15.957 | 16.238 | 224.64 | <0.001 |
| Hypertension | -0.668 | 0.083 | -0.831 | -0.506 | -8.048 | <0.001 |
| Sex | -0.928 | 0.101 | -1.125 | -0.731 | -9.226 | <0.001 |
| Age | -0.117 | 0.003 | -0.123 | -0.111 | -38.517 | <0.001 |
| Age^2^ | -0.004 | <0.001 | -0.004 | -0.003 | -16.608 | <0.001 |
| Education | 0.585 | 0.010 | 0.566 | 0.604 | 59.964 | <0.001 |
| 2‑way: Hypertension * Sex | 0.145 | 0.128 | -0.107 | 0.396 | 1.126 | 0.260 |
| Simple Effect: Yes – No, F | -0.668 | 0.083 | -0.831 | -0.506 | -8.048 | <0.001 |
| Simple Effect: Yes – No, M | -0.524 | 0.100 | -0.719 | -0.328 | -5.255 | <0.001 |
| **BMI** |  |  |  |  |  |  |
| Intercept | 15.701 | 0.279 | 15.154 | 16.249 | 56.225 | <0.001 |
| BMI | 0.001 | 0.009 | -0.017 | 0.018 | 0.066 | 0.947 |
| Sex | -1.998 | 0.503 | -2.985 | -1.012 | -3.971 | <0.001 |
| Age | -0.115 | 0.005 | -0.124 | -0.106 | -24.705 | <0.001 |
| Age^2^ | -0.003 | <0.001 | -0.004 | -0.002 | -8.348 | <0.001 |
| Education | 0.588 | 0.015 | 0.559 | 0.617 | 39.926 | <0.001 |
| 2‑way: BMI * Sex | 0.039 | 0.017 | 0.006 | 0.072 | 2.311 | 0.021 |
| Slope in Women | 0.001 | 0.009 | -0.017 | 0.018 | 0.066 | 0.947 |
| Slope in Men | 0.040 | 0.014 | 0.011 | 0.068 | 2.758 | 0.006 |
| **Alcohol Use** |  |  |  |  |  |  |
| Intercept | 16.276 | 0.091 | 16.097 | 16.456 | 177.996 | <0.001 |
| Alcohol Use | -0.129 | 0.220 | -0.561 | 0.304 | -0.584 | 0.559 |
| Sex | -1.078 | 0.109 | -1.293 | -0.864 | -9.852 | <0.001 |
| Age | -0.117 | 0.005 | -0.127 | -0.107 | -23.319 | <0.001 |
| Age^2^ | -0.004 | <0.001 | -0.004 | -0.003 | -9.064 | <0.001 |
| Education | 0.545 | 0.018 | 0.510 | 0.580 | 30.609 | <0.001 |
| 2‑way: Alcohol Use * Sex | -0.082 | 0.276 | -0.623 | 0.459 | -0.298 | 0.766 |
| Simple Effect: Yes – No, F | -0.129 | 0.220 | -0.561 | 0.304 | -0.584 | 0.559 |
| Simple Effect: Yes – No, M | -0.211 | 0.166 | -0.536 | 0.115 | -1.270 | 0.204 |
| **Social Isolation** |  |  |  |  |  |  |
| Intercept | 15.953 | 0.086 | 15.784 | 16.121 | 185.836 | <0.001 |
| Social Isolation | 0.243 | 0.193 | -0.136 | 0.622 | 1.259 | 0.208 |
| Sex | -1.136 | 0.116 | -1.364 | -0.908 | -9.757 | <0.001 |
| Age | -0.104 | 0.006 | -0.115 | -0.093 | -18.508 | <0.001 |
| Age^2^ | -0.004 | <0.001 | -0.004 | -0.003 | -7.722 | <0.001 |
| Education | 0.585 | 0.018 | 0.550 | 0.621 | 32.161 | <0.001 |
| 2‑way: Social Isolation * Sex | 0.268 | 0.318 | -0.355 | 0.891 | 0.844 | 0.399 |
| Simple Effect: Yes – No, F | 0.243 | 0.193 | -0.136 | 0.622 | 1.259 | 0.208 |
| Simple Effect: Yes – No, M | 0.511 | 0.253 | 0.016 | 1.006 | 2.025 | 0.043 |
| **Poor Vision** |  |  |  |  |  |  |
| Intercept | 15.756 | 0.057 | 15.644 | 15.868 | 275.097 | <0.001 |
| Poor Vision | -0.283 | 0.103 | -0.485 | -0.080 | -2.740 | 0.006 |
| Sex | -0.873 | 0.083 | -1.035 | -0.710 | -10.541 | <0.001 |
| Age | -0.131 | 0.005 | -0.140 | -0.121 | -27.68 | <0.001 |
| Age^2^ | -0.003 | <0.001 | -0.004 | -0.003 | -10.73 | <0.001 |
| Education | 0.593 | 0.011 | 0.572 | 0.613 | 55.456 | <0.001 |
| 2‑way: Poor Vision * Sex | 0.235 | 0.156 | -0.070 | 0.540 | 1.509 | 0.131 |
| Simple Effect: Yes – No, F | -0.283 | 0.103 | -0.485 | -0.080 | -2.740 | 0.006 |
| Simple Effect: Yes – No, M | -0.048 | 0.126 | -0.294 | 0.198 | -0.381 | 0.703 |
| **Poor Sleep** |  |  |  |  |  |  |
| Intercept | 15.751 | 0.059 | 15.751 | 0.059 | 268.105 | <0.001 |
| Poor Sleep | -0.246 | 0.080 | -0.246 | 0.080 | -3.082 | 0.002 |
| Sex | -0.870 | 0.082 | -0.87 | 0.082 | -10.628 | <0.001 |
| Age | -0.124 | 0.003 | -0.124 | 0.003 | -41.463 | <0.001 |
| Age^2^ | -0.004 | <0.001 | -0.004 | <-0.001 | -15.562 | <0.001 |
| Education | 0.590 | 0.010 | 0.590 | 0.010 | 60.639 | <0.001 |
| 2‑way: Poor Sleep * Sex | 0.084 | 0.127 | 0.084 | 0.127 | 0.666 | 0.505 |
| Simple Effect: Yes – No, F | -0.246 | 0.080 | -0.402 | -0.090 | -3.082 | 0.002 |
| Simple Effect: Yes – No, M | -0.162 | 0.099 | -0.356 | 0.033 | -1.630 | 0.103 |
| **Composite Risk Score** |  |  |  |  |  |  |
| Intercept | 16.890 | 0.091 | 15.635 | 15.866 | 185.833 | <0.001 |
| Composite Risk Score | -0.362 | 0.023 | -0.402 | -0.090 | -16.019 | <0.001 |
| Sex | -1.319 | 0.134 | -1.030 | -0.709 | -9.808 | <0.001 |
| Age | -0.117 | 0.003 | -0.130 | -0.118 | -39.304 | <0.001 |
| Age^2^ | -0.004 | <0.001 | -0.004 | -0.003 | -16.261 | <0.001 |
| Education | 0.532 | 0.010 | 0.571 | 0.609 | 51.672 | <0.001 |
| 2‑way: Composite Risk Score * Sex | 0.146 | 0.035 | -0.164 | 0.333 | 4.144 | <0.001 |
| Slope in Women | -0.362 | 0.023 | -0.407 | -0.318 | -16.019 | <0.001 |
| Slope in Men | -0.216 | 0.029 | -0.273 | -0.160 | -7.491 | <0.001 |
| **Adjusted Composite Risk Score** |  |  |  |  |  |  |
| Intercept | 16.566 | 0.082 | 16.406 | 16.727 | 202.634 | <0.001 |
| Adjusted Composite Risk Score | -3.680 | 0.267 | -4.202 | -3.158 | -13.806 | <0.001 |
| Sex | -1.269 | 0.123 | -1.51 | -1.029 | -10.345 | <0.001 |
| Age | -0.117 | 0.003 | -0.123 | -0.111 | -39.063 | <0.001 |
| Age^2^ | -0.004 | <0.001 | -0.004 | -0.003 | -15.934 | <0.001 |
| Education | 0.539 | 0.011 | 0.518 | 0.559 | 51.262 | <0.001 |
| 2‑way: Adjusted Composite Risk Score * Sex | 1.870 | 0.416 | 1.054 | 2.686 | 4.492 | <0.001 |
| Slope in Women | -3.68 | 0.267 | -4.202 | -3.158 | -13.806 | <0.001 |
| Slope in Men | -1.81 | 0.349 | -2.494 | -1.126 | -5.186 | <0.001 |

Complete statistics for all exploratory Firth logistic regressions examining two-way interactions between the presence of risk factor at baseline and sex on cognitive status (unimpaired vs. impaired) 8 years later, stratified by age group, for each of the 13 risk factors assessed are presented below. Significant two-way interactions were further stratified by risk factor presence to examine the main effect of sex on cognitive status. The significant two-way interaction between hypertension and sex is reported in the body of the manuscript.

For completeness, we also report here that several risk factors increased the risk of cognitive impairment (i.e., a main effect of risk factor), depending on the age group. Within the 40-59 years age group, those with depression had increased odds of cognitive impairment compared to those without depression (OR=2.31, 95% CI [1.09, 4.79], p=.030). Within the 60-79 years age group, those with hearing loss (OR=1.32, 95% CI [1.03, 1.68], p=.028), depression (OR=1.41, 95% CI [1.02, 1.95], p=0.04), diabetes (OR=1.80, 95% CI [1.34, 2.40], p<.001), hypertension (OR=1.49, 95% CI [1.15, 1.94], p=0.003), poor vision (OR=1.58, 95% CI [1.18, 2.11], p=0.003), or poor sleep (OR=1.28, 95% CI [1.00, 1.64], p=0.049) had greater odds of cognitive impairment compared to those without the risk factor.

| **Model Terms** | **Odds Ratio** | **95% CI Lower** | **95% CI Upper** | **p-value** |
| --- | --- | --- | --- | --- |
| **Poor Hearing** |  |  |  |  |
| 40-59 yrs |  |  |  |  |
| Poor Hearing | 0.95 | 0.47 | 1.86 | .871 |
| Sex | 1.18 | 0.58 | 2.33 | .650 |
| Poor Hearing * Sex Interaction | 1.40 | 0.52 | 3.84 | .514 |
| 60-79 yrs |  |  |  |  |
| Poor Hearing | 1.32 | 1.03 | 1.68 | .028 |
| Sex | 1.08 | 0.79 | 1.47 | .634 |
| Poor Hearing * Sex Interaction | 0.78 | 0.52 | 1.73 | .231 |
| 80+ yrs |  |  |  |  |
| Poor Hearing | 0.87 | 0.44 | 1.70 | .313 |
| Sex | 0.27 | 0.08 | 0.86 | .026* |
| Poor Hearing * Sex Interaction | 2.63 | 0.68 | 11.36 | .163 |
| **Depression** |  |  |  |  |
| 40-59 yrs |  |  |  |  |
| Depression | 2.31 | 1.09 | 4.79 | .030 |
| Sex | 1.54 | 0.87 | 2.73 | .137 |
| Depression * Sex Interaction | 0.85 | 0.25 | 2.80 | .794 |
| 60-79 yrs |  |  |  |  |
| Depression | 1.41 | 1.02 | 1.95 | .040 |
| Sex | 1.03 | 0.83 | 1.27 | .816 |
| Depression * Sex Interaction | 0.65 | 0.32 | 1.30 | .229 |
| 80+ yrs |  |  |  |  |
| Depression | 0.61 | 0.20 | 1.78 | .203 |
| Sex | 0.56 | 0.30 | 1.03 | .062 |
| Depression * Sex Interaction | 0.47 | <0.01 | 7.68 | .628 |
| **Physical Inactivity** |  |  |  |  |
| 40-59 yrs |  |  |  |  |
| Physical Inactivity | 0.76 | 0.39 | 1.50 | .643 |
| Sex | 1.28 | 0.56 | 2.85 | .344 |
| Physical Inactivity * Sex Interaction | 1.18 | 0.43 | 3.29 | .097 |
| 60-79 yrs |  |  |  |  |
| Physical Inactivity | 0.85 | 0.66 | 1.09 | .203 |
| Sex | 0.98 | 0.70 | 1.38 | .919 |
| Physical Inactivity * Sex Interaction | 1.00 | 0.65 | 1.53 | .992 |
| 80+ yrs |  |  |  |  |
| Physical Inactivity | 0.85 | 0.76 | 2.35 | .644 |
| Sex | 0.27 | 0.08 | 0.79 | .016* |
| Physical Inactivity * Sex Interaction | 2.92 | 0.79 | 11.66 | .108 |
| **Diabetes** |  |  |  |  |
| 40-59 yrs |  |  |  |  |
| Diabetes | 1.61 | 0.74 | 3.37 | .223 |
| Sex | 1.55 | 0.89 | 2.71 | .118 |
| Diabetes * Sex Interaction | 0.67 | 0.18 | 2.37 | .536 |
| 60-79 yrs |  |  |  |  |
| Diabetes | 1.80 | 1.34 | 2.40 | <.001* |
| Sex | 0.98 | 0.78 | 1.24 | .874 |
| Diabetes * Sex Interaction | 0.85 | 0.54 | 1.36 | .506 |
| 80+ |  |  |  |  |
| Diabetes | 0.87 | 0.35 | 2.20 | .219 |
| Sex | 0.55 | 0.28 | 1.05 | .070 |
| Diabetes * Sex Interaction | 1.00 | 0.20 | 4.77 | .999 |
| **Smoking** |  |  |  |  |
| 40-59 yrs |  |  |  |  |
| Smoking | 0.69 | 0.16 | 2.39 | .571 |
| Sex | 1.87 | 0.81 | 4.56 | .144 |
| Smoking * Sex Interaction | 1.66 | 0.32 | 9.52 | .551 |
| 60-79 yrs |  |  |  |  |
| Smoking | 1.14 | 0.74 | 1.74 | .546 |
| Sex | 1.07 | 0.80 | 1.42 | .650 |
| Smoking * Sex Interaction | 0.60 | 0.30 | 1.21 | .154 |
| 80+ |  |  |  |  |
| Smoking | 1.24 | 0.10 | 16.20 | .855 |
| Sex | 0.74 | 0.39 | 1.75 | .490 |
| Smoking * Sex Interaction | 4.01 | 0.87 | 873.74 | .505 |
| **Hypertension** |  |  |  |  |
| 40-59 yrs |  |  |  |  |
| Hypertension | 0.72 | 0.36 | 1.42 | .349 |
| Sex | 1.15 | 0.57 | 2.29 | .697 |
| Hypertension * Sex Interaction | 1.59 | 0.59 | 4.40 | .366 |
| 60-79 yrs |  |  |  |  |
| Hypertension | 1.49 | 1.15 | 1.94 | .002* |
| Sex | 1.19 | 0.86 | 1.66 | .293 |
| Hypertension * Sex Interaction | 0.75 | 0.50 | 1.14 | .180 |
| 80+ |  |  |  |  |
| Hypertension | 1.76 | 0.85 | 3.67 | .127 |
| Sex | 1.09 | 0.42 | 2.82 | .851 |
| Hypertension * Sex Interaction | 0.33 | 0.10 | 1.14 | .080* |
| Hypertension group: Sex | 0.36 | 0.16 | 0.80 | .011* |
| No hypertension group: Sex | 1.09 | 0.42 | 2.82 | .851 |
| **Alcohol Use** |  |  |  |  |
| 40-59 yrs |  |  |  |  |
| Alcohol Use | 0.78 | 0.78 | 3.92 | .781 |
| Sex | 0.89 | 0.34 | 2.27 | .806 |
| Alcohol Use * Sex Interaction | 2.12 | 0.28 | 26.48 | .487 |
| 60-79 yrs |  |  |  |  |
| Alcohol Use | 1.19 | 0.60 | 2.27 | .607 |
| Sex | 1.02 | 0.70 | 1.47 | .925 |
| Alcohol Use * Sex Interaction | 0.94 | 0.40 | 2.24 | .879 |
| 80+ |  |  |  |  |
| Alcohol Use | 0.92 | 0.18 | 4.76 | .766 |
| Sex | 0.64 | 0.23 | 1.77 | .393 |
| Alcohol Use * Sex Interaction | 1.55 | 0.08 | 32.13 | .765 |
| **Social Isolation** |  |  |  |  |
| 40-59 yrs |  |  |  |  |
| Social Isolation | 0.33 | 0.18 | 0.58 | .140 |
| Sex | 0.67 | 0.24 | 1.75 | .420 |
| Social Isolation * Sex Interaction | 14.06 | 0.75 | 2248.90 | .080 |
| 60-79 yrs |  |  |  |  |
| Social Isolation | 1.02 | 0.49 | 1.98 | .967 |
| Sex | 0.95 | 0.65 | 1.37 | .786 |
| Social Isolation * Sex Interaction | 1.79 | 0.63 | 5.11 | .271 |
| 80+ |  |  |  |  |
| Social Isolation | 0.07 | <0.01 | 0.63 | .013 |
| Sex | 1.00 | 0.29 | 3.44 | 1.000 |
| Social Isolation * Sex Interaction | 10.00 | 0.43 | 2999.12 | .136 |
| **Poor Vision** |  |  |  |  |
| 40-59 yrs |  |  |  |  |
| Poor Vision | - | - | - | - |
| Sex | - | - | - | - |
| Poor Vision * Sex Interaction | - | - | - | - |
| 60-79 yrs |  |  |  |  |
| Poor Vision | 1.58 | 1.18 | 2.11 | .003 |
| Sex | 0.97 | 0.77 | 1.22 | .814 |
| Poor Vision * Sex Interaction | 1.12 | 0.68 | 1.84 | .648 |
| 80+ |  |  |  |  |
| Poor Vision | 1.24 | 0.63 | 2.44 | .801 |
| Sex | 0.83 | 0.36 | 1.93 | .539 |
| Poor Vision * Sex Interaction | 0.47 | 0.14 | 1.54 | .210 |
| **Poor Sleep** |  |  |  |  |
| 40-59 yrs |  |  |  |  |
| Poor Sleep | 0.87 | 0.45 | 1.70 | .678 |
| Sex | 1.47 | 0.75 | 2.91 | .258 |
| Poor Sleep * Sex Interaction | 0.84 | 0.31 | 2.26 | .728 |
| 60-79 yrs |  |  |  |  |
| Poor Sleep | 1.28 | 1.00 | 1.64 | .049 |
| Sex | 1.04 | 0.80 | 1.34 | .771 |
| Poor Sleep * Sex Interaction | 0.87 | 0.57 | 1.30 | .493 |
| 80+ |  |  |  |  |
| Poor Sleep | 0.72 | 0.35 | 1.48 | .369 |
| Sex | 0.58 | 0.28 | 1.18 | .130 |
| Poor Sleep * Sex Interaction | 0.94 | 0.26 | 3.35 | .926 |
| **Model Terms (continuous variables)** | **Odds Ratio** | **95% CI Lower** | **95% CI Upper** | **p-value** |
| **Education** |  |  |  |  |
| 40-59 yrs |  |  |  |  |
| Education | 0.91 | 0.82 | 1.01 | .080 |
| Sex | 2.25 | 0.31 | 17.24 | .426 |
| Education * Sex Interaction | 0.97 | 0.83 | 1.12 | .642 |
| 60-79 yrs |  |  |  |  |
| Education | 0.96 | 0.92 | 1.01 | .093 |
| Sex | 0.45 | 0.17 | 1.21 | .113 |
| Education * Sex Interaction | 1.06 | 0.99 | 1.14 | .114 |
| 80+ |  |  |  |  |
| Education | 1.01 | 0.88 | 1.16 | .893 |
| Sex | 0.28 | 0.01 | 5.36 | .398 |
| Education * Sex Interaction | 1.05 | 0.85 | 1.32 | .643 |
| **Total Cholesterol** |  |  |  |  |
| 40-59 yrs |  |  |  |  |
| Total Cholesterol | 1.00 | 0.98 | 1.01 | 0.691 |
| Sex | 1.04 | 0.01 | 86.47 | 0.985 |
| Total Cholesterol * Sex Interaction | 1.00 | 0.98 | 1.02 | 0.904 |
| 60-79 yrs |  |  |  |  |
| Total Cholesterol | 1.00 | 0.98 | 1.01 | 0.598 |
| Sex | 4.02 | 0.82 | 20.08 | 0.086 |
| Total Cholesterol * Sex Interaction | 0.99 | 0.99 | 1.00 | 0.095 |
| 80+ |  |  |  |  |
| Total Cholesterol | 1.01 | 0.98 | 1.03 | 0.106 |
| Sex | 0.21 | 0.00 | 60.16 | 0.592 |
| Total Cholesterol * Sex Interaction | 1.01 | 0.98 | 1.04 | 0.574 |
| **Obesity** |  |  |  |  |
| 40-59 yrs |  |  |  |  |
| BMI | 1.01 | 0.93 | 1.08 | .893 |
| Sex | 12.97 | 0.10 | 2391.59 | .308 |
| BMI * Sex Interaction | 0.93 | 0.78 | 1.10 | .404 |
| 60-79 yrs |  |  |  |  |
| BMI | 0.99 | 0.96 | 1.02 | .576 |
| Sex | 0.49 | 0.09 | 2.62 | .403 |
| BMI * Sex Interaction | 1.03 | 0.97 | 1.09 | .369 |
| 80+ |  |  |  |  |
| BMI | 1.00 | 0.89 | 1.13 | .973 |
| Sex | 4.08 | 0.04 | 816.20 | .567 |
| BMI * Sex Interaction | 0.95 | 0.78 | 1.13 | .532 |
| P-values for t-test comparisons reflect values uncorrected for multiple comparisons due to the exploratory nature of this analysis.  Women were the reference group for the main effect of sex. | | | | |
